# Pore “Softening” and Emergence of Breathability Effects of New Keplerate Nano‐Containers

**DOI:** 10.1002/anie.202218897

**Published:** 2023-03-09

**Authors:** Alexander Elliott, James McAllister, De‐Liang Long, Yu‐Fei Song, Haralampos N. Miras

**Affiliations:** ^1^ School of Chemistry The University of Glasgow Glasgow G12 8QQ UK; ^2^ State Key Laboratory of Chemical Resource Engineering Beijing Advanced Innovation Centre for Soft Matter Science and Engineering Beijing University of Chemical Technology Beijing 100029 China

**Keywords:** Host-Guest Systems, Keplerate, Polyoxometalates, Selenium, Self-Assembly

## Abstract

The self‐assembly of porous molecular nanocapsules offer unique opportunities to investigate a range of interesting phenomena and applications. However, to design nanocapsules with pre‐defined properties, thorough understanding of their structure‐property relation is required. Here, we report the self‐assembly of two elusive members of the Keplerate family, [Mo_132_Se_60_O_312_(H_2_O)_72_(AcO)_30_]^42−^ {Mo_132_Se_60_} **1** and [W_72_Mo_60_Se_60_O_312_(H_2_O)_72_(AcO)_30_]^42−^ {W_72_Mo_60_Se_60_} **2**, that have been synthesised using pentagonal and dimeric ([Mo_2_O_2_Se_2_]^2+^) building blocks and their structures have been confirmed via single crystal X‐ray diffractions. Our comparative study involving the uptake of organic ions and the related ligand exchange of various ligand sizes by the {Mo_132_Se_60_} and previously reported Keplerates {Mo_132_O_60_}, {Mo_132_S_60_} based on the ligand exchange rates, revealed the emergence of increased “breathability” that dominates over the pore size as we transition from the {Mo_132_S_60_} to the “softer” {Mo_132_Se_60_} molecular nano‐container.

## Graphical abstract

Engineering the porosity of new Keplerate type molecular nano‐containers for the controlled interaction with their external chemical environment is investigated. Their crystal structure has been determined by single crystal X‐ray diffractions while the balancing act of kinetic and thermodynamic factors in conjunction with the emergence of amplified pore breathability that govern the functionality of the nano‐containers is discussed.

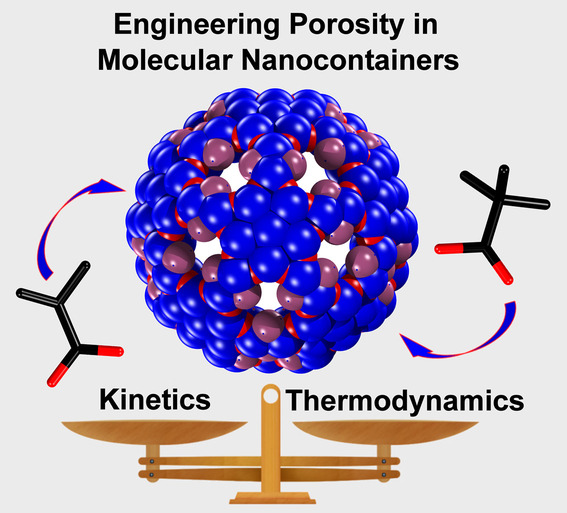

## Introduction

Since it was first reported in 1998 by Müller^[1]^ the “Keplerate” structure [Mo_132_O_372_(H_2_O)_72_(AcO)_30_]^42−^ {Mo_132_O_60_} has proved a structurally robust family of high nuclearity inorganic fullerene‐type molecules. Consisting of 12 {Mo_6_} pentagonal units linked by 30 metal ions or {Mo_2_} linker units, the structure may be obtained from a wide variety of building blocks while retaining its overall topology. In the original structure these consist of {Mo^VI^Mo^VI^_5_} pentagonal units linked by [Mo^V^_2_O_4_]^2+^ bridges, however the pentagonal units can be exchanged for tungstate equivalents,[^2^, ^3^] whilst a wide range of bridges including [Mo^V^_2_S_2_O_2_]^2+[4, 5]^ (therefore {Mo_132_S_60_}) and single Fe^III^,^[6]^ V^IV^O^[7]^ and Cr^III[8]^ units have been employed leading effectively not only to the sizing of molybdenum‐ oxide‐based molecular icosahedra but to the engineering of porosity.

Most importantly, these molybdenum oxide nano‐containers can interact with their chemical environment. They incorporate crown ether‐type {Mo_9_O_9_} pores that can interact either via hydrogen bonding with organic cations,[^9^, ^10^, ^11^] with metal ions,[^12^, ^13^] or via steric hindrance imposed by the dimensions of the pore.

When dimeric bridges such as [Mo^V^_2_O_4_]^2+^ are used, a distorted truncated icosahedron is achieved^[14]^ with large hexagonal faces bounded on three sides by pentagonal units, and on the remaining three sides by dimeric bridges. These hexagonal faces exhibit large pores that can allow guests to enter the large central cavity of the Keplerate. When [Mo^V^_2_O_4_]^2+^ is used as the linker the internal face of the Keplerate is lined with ligands attached to the dimeric bridges. Standard synthetic methods performed in acetate buffer produce a Keplerate lined with acetate ligands, making the interior less hydrophilic than the exterior. These internal ligands however are highly labile, and encapsulated anionic guests possessing the appropriate bidentate geometry can exchange with internal ligands, allowing a range of post‐synthetic modifications to take place.[^15^, ^16^] The host properties of the Keplerate combined with tuning of the internal cavity via replacement of acetate with alternative ligands has found diverse uses as a nanoreactor,[^17^, ^18^] in chiral recognition^[19]^ and as a potential drug delivery mechanism.^[20]^

It has been observed initially that the maximum size of ligand that may be exchanged is dictated by its ability to pass through the open pores, with smaller ligands exchanging faster, and larger ligands being excluded entirely.^[21]^ Consequently, controlling the pore size through modifications to the Keplerate, the molecules that are excluded from the central cavity of the Keplerate could be controlled in a tuneable manner.^[9]^ When ligands are present in solution as well as within the Keplerate a dynamic equilibrium is achieved^[15]^ between the ligands inside and outside of the Keplerate, when the Keplerate is instead placed in pure water the ligands inside the Keplerate leach out. This may lead to a loss of functionality where functional ligands are used and may lead to disintegration of the Keplerate without the stabilising influence of the ligands.^[22a]^ Loss of ligands may be avoided by fully blocking the pore with guanidinium ([C(NH_2_)_3_]^+^) cations, however this fully blocks the pore preventing the entry of potential guests. It was envisaged that selection of internal ligands of appropriate size can prevent them from leaching out of the nano‐container, whilst allowing smaller molecules to enter the Keplerate as guests. To do so, pore size must be controlled to balance the inhibition of the escape of internal ligands against kinetically feasible entrance of desired guests into the structure. Linkers such as [Mo^V^_2_S_2_O_2_]^2+^ offer coordination sites for internal ligands whilst also shrinking the pore due to the greater size of the sulphide anion compared to [Mo^V^_2_O_4_]^2+^ oxide‐based linkers but have so far not been studied in terms of exchange kinetics.

Here in we report firstly the self‐assembly of two new elusive members of the Keplerate family, [Mo_132_Se_60_O_312_(H_2_O)_72_(AcO)_30_]^42−^ {Mo_132_Se_60_} **1**, and [W_72_Mo_60_Se_60_O_312_(H_2_O)_72_(AcO)_30_]^42−^ {W_72_Mo_60_Se_60_} **2**, that possess “softer” pores by the incorporation of the Se^2−^ anion in the form of [Mo^V^_2_O_2_Se_2_]^2+^ linker units to molybdenum as well as tungsten‐based Keplerates. The selenium‐based linker further restricts the pore size compared to [Mo^V^_2_O_4_]^2+^ and [Mo^V^_2_O_2_S_2_]^2+^ linked Keplerates and thereby offers an additional design option when controlling pore size. The syntheses and crystallographic characterization proved to be extremely challenging tasks. The inherent instability of the required Se‐based constituents used to construct the “softer” nanosized Keplerates and subsequent isolation of single crystals of sufficient quality is a testament of the reason that these species remained elusive for very long time. Comparison of exchange kinetics of the three Keplerate nano‐containers revealed a disruption of the expected trend based on the shrinking pore size as we move from the larger pore size of {Mo_132_} to the smaller ones observed in {Mo_132_S_60_} by an unexpected emergence of an increased pore breathability as we move to the {Mo_132_Se_60_} Keplerate where the pore flexibility appears to be the dominant factor on the observed exchange behaviour.

## Results and Discussion

### Molecular structure

**Figure 1:**
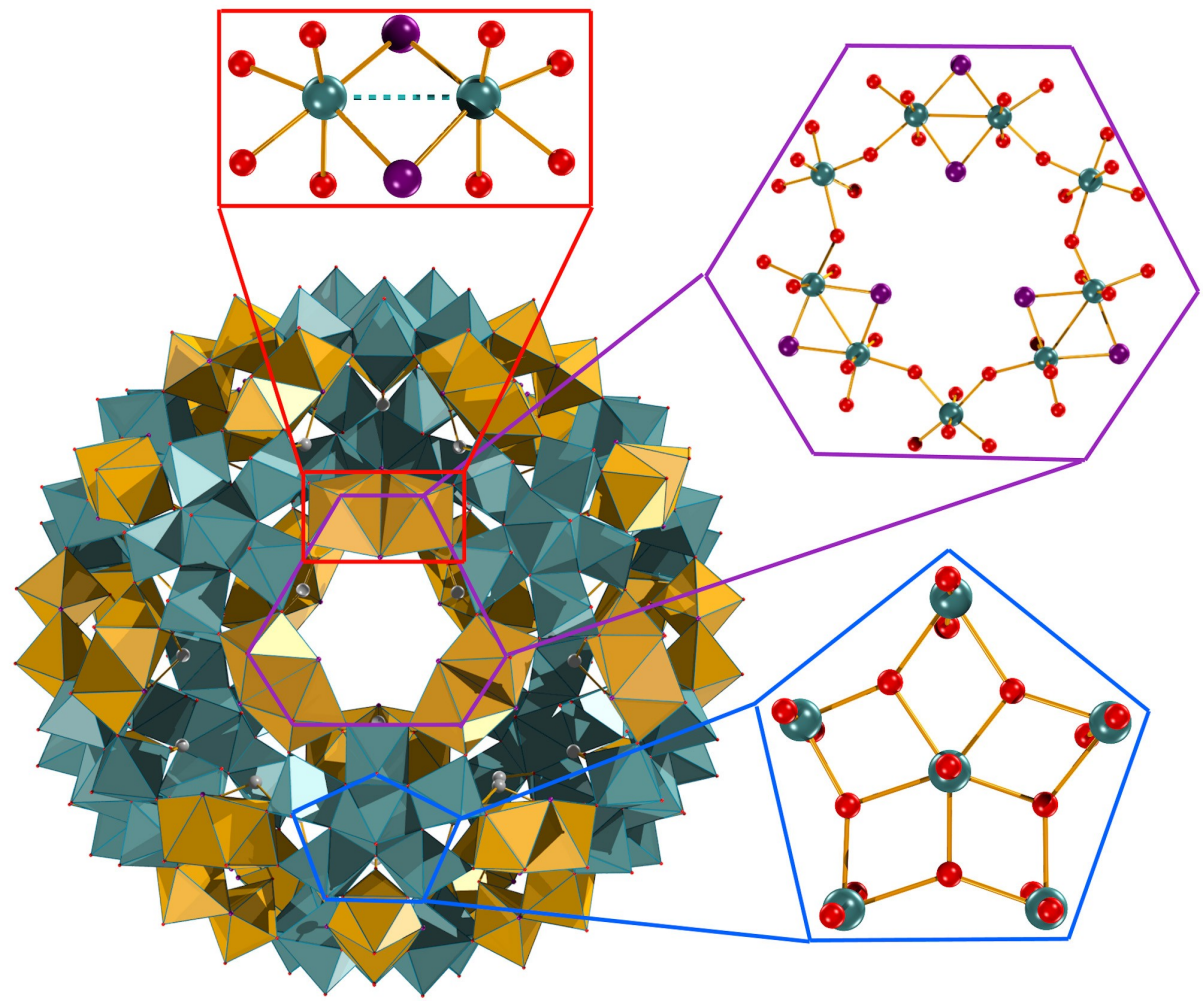
Polyhedral representation of [Mo_132_Se_60_O_312_(H_2_O)_72_(AcO)_30_]^42−^ structure and its building blocks (hydrogen atoms omitted for clarity); pentagonal [Mo^VI^_6_O_21_(H_2_O)_6_]^6−^ units (Teal polyhedra), dimeric [Mo_2_O_2_Se_2_(AcO)]^+^ units (Yellow polyhedra). The hexagonal pores formed are indicated in purple. Ball‐and‐Stick colour code: Mo, teal; Se, purple; Oxygen, red; Carbon, white. Crystallographic parameters of **1** and **2** are summarised in Table S1.

The key parameter in the discovery of the new nano‐containers was the preparation of the [Mo^V^_2_O_2_Se_2_]^2+^ dimeric unit in a stable form that could be incorporated into the Keplerate architecture, Figure 1, in an analogous manner as reported by Cadot *et al* ^[4]^ for the sulphide equivalent [Mo^V^_2_O_2_S_2_]^2+^. Recrystallisation with ammonium chloride yielded hexagonal plate‐like crystals suitable for X‐ray diffraction studies from which a definitive structural determination confirmed the presence of 30 [Mo^V^_2_O_2_Se_2_]^2+^ units linking the twelve pentagonal [Mo^VI^_6_O_21_]^6−^ building blocks into an “{Mo_132_Se_60_}” nanosized icosahedral moiety. The final structure has an approximate formula of [Mo_132_Se_60_O_312_(H_2_O)_72_(AcO)_30_]^42−^ (discounting counter cations and disordered water inside and outside the icosahedron) (**1**)

Substitution of sodium molybdate for sodium tungstate led to the formation and isolation of an isostructural tungsten based Keplerate ({W_72_Mo_60_Se_60_}) (**2**). Recrystallization in the presence of ammonium chloride yielded large block shaped crystals of **2**. Pleasantly, we were able to determine the crystal structure of **2** even though the X‐ray crystallographic work is highly challenging with heavy disorder observed over Mo and W sites in the cubic symmetrical system. We note similar challenges faced in the structural determination of {W_72_Mo_60_}^[22b]^ and {W_72_Mo_60_S_60_}.^[5]^

In this way a series of three otherwise structurally equivalent Keplerates could be synthesised, the already reported {Mo_132_S_60_}^[4]^ (**3**), {Mo_132_O_60_}^[1]^ (**4**), and new {Mo_132_Se_60_} (**1**), differing only in the chalcogenide present linking the Mo^V^ dimeric linkers. This gives similar overall structural properties except for the pore dimensions. Whereas the oxygen Keplerate features a O_9_ pore bounded by nine oxygen atoms, the three closest oxygen atoms are replaced for sulphur or selenium, giving {S_3_O_6_}, and {Se_3_O_6_} pore types respectively. As the chalcogen increases in size the total pore area becomes less symmetrical, with the outer six oxygen atoms playing an increasingly smaller part in defining the accessible pore area. The Mo^V^−Mo^V^ bond length increases O<S<Se (2.611(6),^[1]^ 2.815(2)^[4]^ and 2.868(9) Å) leading to an expansion of the diameter of the Keplerate (average pentagon‐pentagon spacing is 25.69(1), 26.0(9) and 26.2(2) Å^[23]^ for O, Se and S respectively) however at the same time the increase in chalcogenide size restricts the size of the pore opening (Table 1). Three chalcogenides reach closest to the centre of the pore, and thereby determine the maximum size of moieties allowed to pass through the shrinking pores. The spacing between chalcogenides decreases O>S>Se (5.71(3), 5.16(5) and 4.89(5) Å respectively), while the increase in Van der Waals radius of the chalcogenides leads to a significant decrease in the pore radius (minimum distance between an atom edge and centre of the pore) O≫S>Se (1.78, 1.17 and 0.94 Å). Except for the stark differences in pore diameters, all other structural parameters remain unchanged between the three nano‐containers with an approximate 6 % difference in volume between the largest and smallest nanocontainer (based on the change in radius). The drastic changes of pore size should greatly affect the rate at which guests enter the cavity of the Keplerate when acting as a host.

**Table 1:** Important distances in {Mo_132_O_60_}, {Mo_132_S_60_} and {Mo_132_Se_60_} Keplerates. E−E bond lengths are averaged for all pores, the pore radius is then calculated based on the van der Waals radii of the chalcogen, and the icosahedron radius is based on the average Mo^VI^−Mo^VI^ distance between centres of pentagonal units on opposite sides of each molecular icosahedron (Figure S1).

	E−E distance [Å]	Pore radius [Å]	Diameter of icosahedron [Å]
**1** {Mo_132_Se_60_}	4.89(5)	0.94	26.0(9)
**3** {Mo_132_S_60_}	5.16(5)	1.17	26.2(2)
**4** {Mo_132_O_60_}	5.71(3)	1.78	25.7(1)

### Single ligand exchange

**Figure 2:**
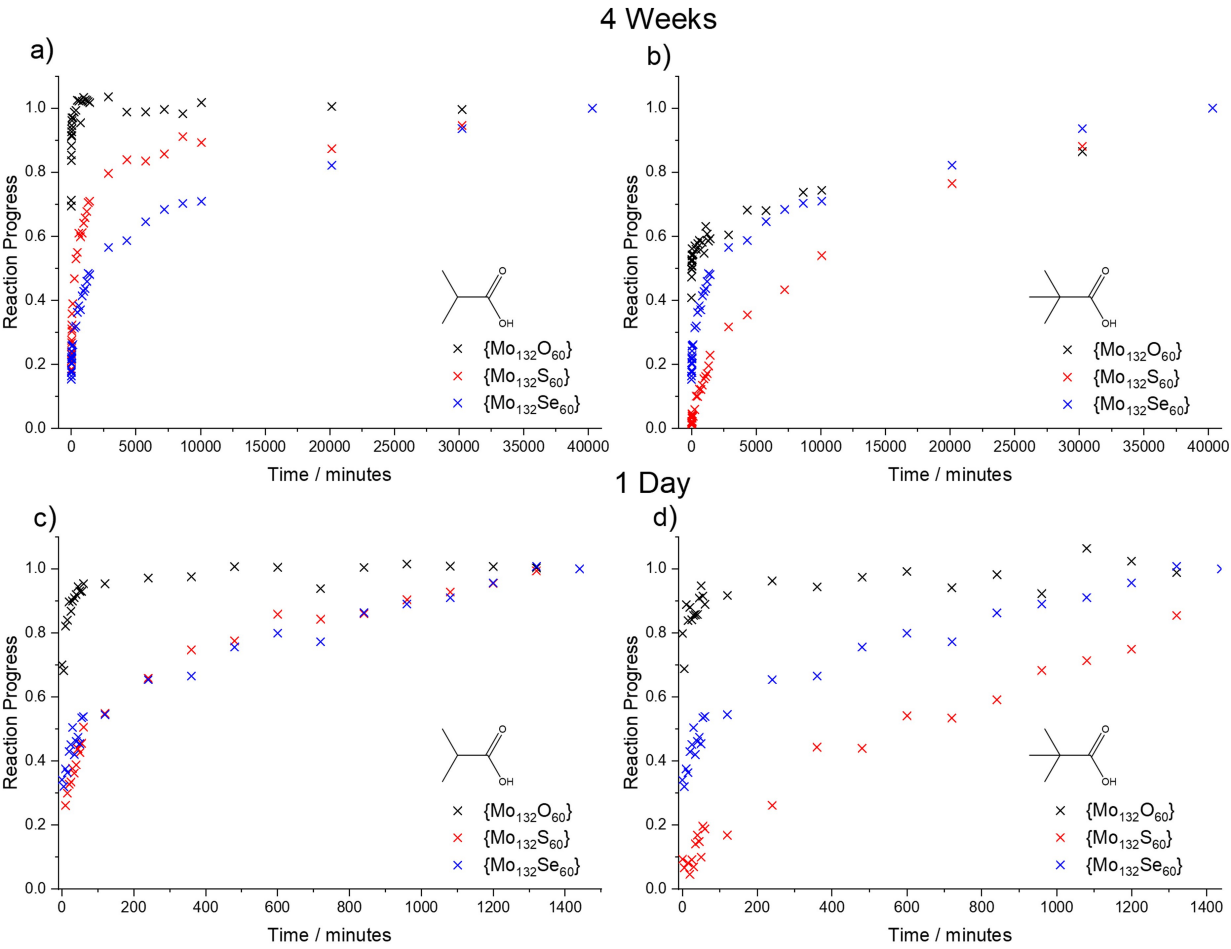
Fraction of final number of exchanged ligands over time for {Mo_132_O_60_} (black), {Mo_132_S_60_} (red) and {Mo_132_Se_60_} (blue) Keplerates exchanging with a) isobutyric acid b) pivalic acid over the course of 4 weeks and c) isobutyric acid d) pivalic acid over the course of one day. The difference between the rates of exchange of isobutyric acid in {Mo_132_S_60_} and {Mo_132_Se_60_} Keplerates is only obvious at the longer timescale, however the low stability of the {Mo_132_Se_60_} capsule over the course of 4 weeks means that the initial rate may be exaggerated at this timescale when comparing rates of pivalic acid exchange, but at the timescale of one day represents accurately the system.

**Figure 3:**
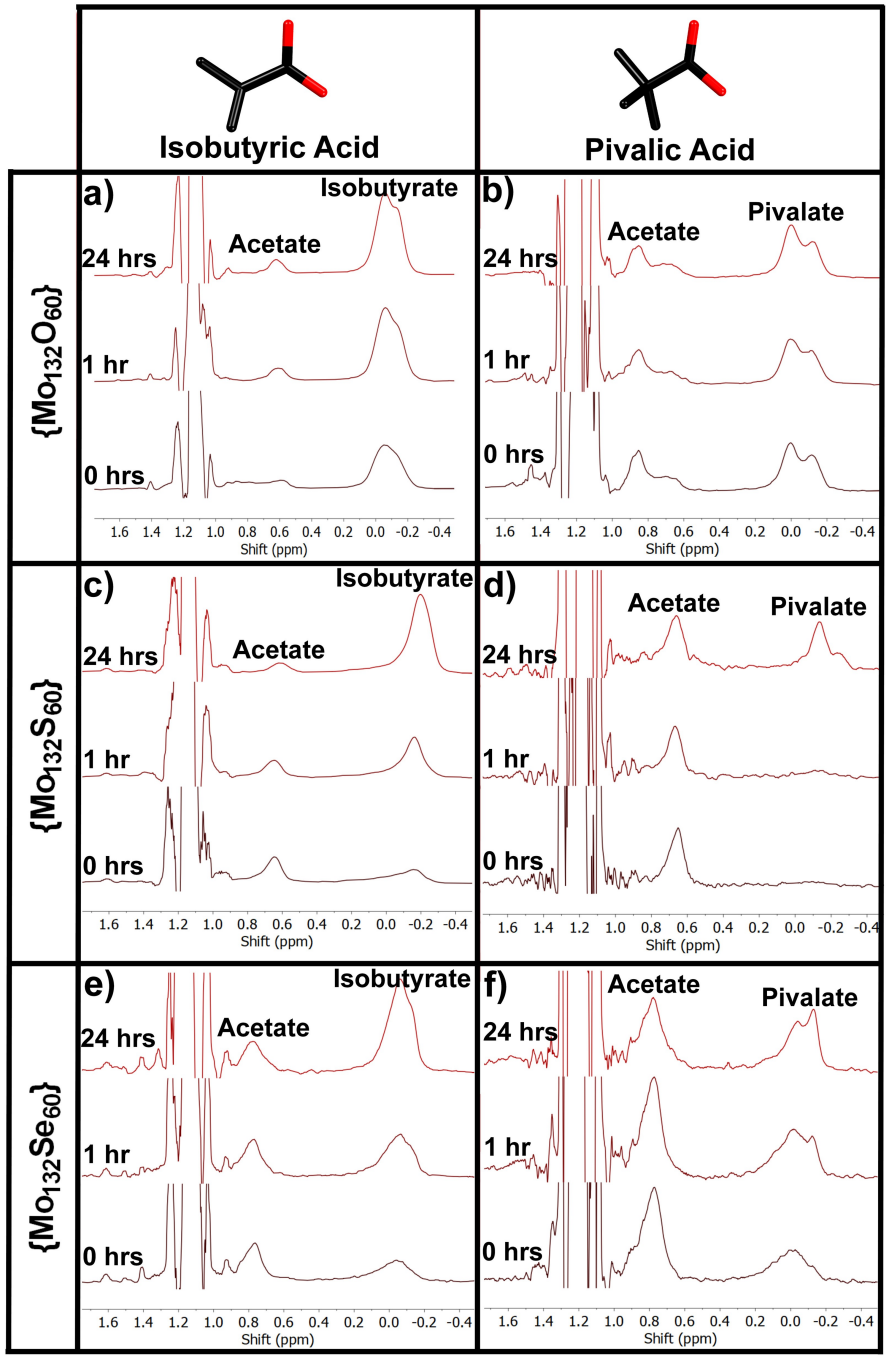
^1^H NMR spectra immediately after mixing, after one hour and after 24 hours for a) {Mo_132_O_60_} **4**, b) {Mo_132_S_60_} **3** and c) {Mo_132_Se_60_} **1** Keplerates in the presence of isobutyric acid and d) {Mo_132_O_60_} **4**, e) {Mo_132_S_60_} **3** and f) {Mo_132_Se_60_} **1** Keplerates in the presence of pivalic acid. In each case the peak at 0.7–0.8 ppm belongs to acetate initially present within the structure, and the growing peak at 0 ppm belongs to the exchanging acid. Selenide shows the acetate peak at slightly (0.1 ppm) higher shift due to decreased donation of charge from the acetate to the metal.

As a model for other, similarly sized guests, RCOOH carboxylate ligands were used to assess the rate at which guests of different sizes could enter the internal cavity (Figure 2). When R is an alkyl chain the difference in electronic effects of bonding is not expected to be significant (p*K*_a_ of acetic acid is 4.75 while the p*K*_a_ of propionic, isobutyric and pivalic acids are 4.87, 4.87 and 4.93, respectively). ^1^H NMR proved a straightforward method to monitor the progression of the ligand exchange (Figures 3 and S3). The methyl peak present in the ligands studied can be easily distinguished from other peaks and shifts from 1 ppm when free to a broad double peak between −0.35 ppm and 0.16 ppm (Figure 3). Notably both the encapsulated and the free methyl proton peaks lie significantly upfield of the equivalent methyl protons belonging to the acetate ligands initially present and appear distinct from all other signals in the spectrum allowing an easy quantification. The double peak, which is also present in encapsulated acetate protons is believed to arise based on the occupancy of secondary sites on pentagonal units by either acetate or the guest ligand.^[10]^

Diffusion ordered spectroscopy (DOSY) was used to verify the assignments of these peaks. Encapsulated guest ligands showed an approximately 8‐fold decrease in diffusion rate compared to the free ligand, whilst acetate showed an approximately 12‐fold decrease, which strongly agrees with the assignment of these peaks as arising from ligands which are encapsulated within the Keplerate (Figure S4).

To confirm that steric hindrance of transfer across the pore barrier was the rate determining step of ligand exchange, the straight chain propionic acid (R=CH_2_CH_3_) was tested showing very rapid uptake (near complete replacement of the acetate signal in approximately 10 minutes) in the absence of steric hindrance. In subsequent experiments the bulkier isobutyric (R=CH(CH_3_)_2_) and pivalic (R=C(CH_3_)_3_) acid were used showing much slower uptake, indicating that the rate of uptake for these ligands is largely based on steric considerations since the electronic properties should be very similar between these different ligands. Lastly to avoid issues of different equilibria between different ligands due either to thermodynamic considerations or steric hindrance *inside* the Keplerate the assessment of rate should be restricted to the initial rate of uptake.

As expected, the rate of exchange is initially fast and decreases over time as an equilibrium is approached (Figure 2). The bulkier Pivalic acid (R=C(CH_3_)_3_) consistently exchanges slower than the smaller isobutyric acid (R=CH(CH_3_)_2_) for all three capsules. This is in agreement with previous reports by Weinstock et al. on the exchange for {Mo_132_O_60_} Keplerates.^[15]^

Isobutyric acid (R=CH(CH_3_)_2_) showed fastest exchange with **4**, followed by **3** and then **1** as expected based on the relative pore sizes. Pivalic acid (R=C(CH_3_)_3_) on the other hand shows a partial inversion of this order in which **1** exchanges faster than **3** initially, whilst both exchange slower than **4**. This counterintuitive result suggests that effects other than the rigid pore size govern the rate of ligand transport.

To discount the possibility that the apparent rapid approach of equilibrium could be attributed to the breakdown of **1** whilst in solution we studied the stability of the structure using UV/Vis (Figure S1) which showed **1** is stable during the course of the investigation (See Supporting Information). Further to this the relative rate of capsule disintegration appears slow based on NMR as in each experiment the fraction of encapsulated ligands continued to rise over the course of the entire 4 weeks studied. Even after 4 weeks evidence of instability is negligible, therefore the instability may be discounted as the cause of this phenomenon.

**Figure 4:**
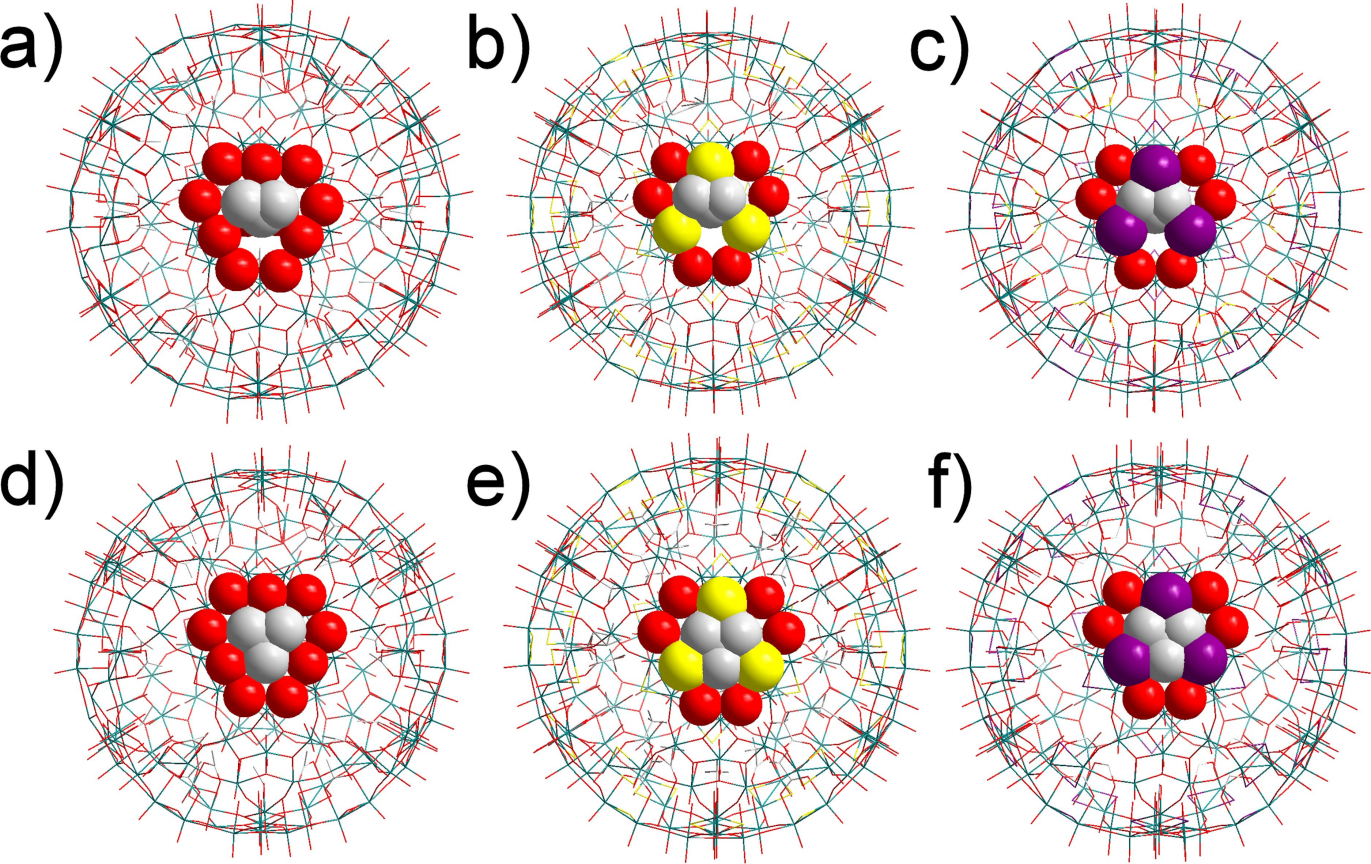
Modelled depiction of ligand structures overlayed on Keplerates based on their crystal structure. Isobutyric (a, b, and c) and pivalic (d, e, and f) acids placed inside the pores of **4** (a and d), **3** (b and e) and **1** (c and f). Ligands and pore are shown as space filling spheres based on their Van der Waals radii, other atoms are shown as vertexes between stick bonds. Ligands are placed such that methyl groups lie in the plane of the pore and are then rotated and translated within the plane to minimise overlap of van der Waals radii. Carbon shown as white, oxygen as red, sulphur as yellow, selenium as purple and molybdenum as teal. Hydrogens are omitted for clarity.

Based solely on pore dimensions arguments, in no structure is it possible for pivalic acid to pass through the pores without intersecting Van der Waals radius between the ligand and the pore boundaries (Figure 4). Even though the distortion of the pore required for **4** is very minor, it increases significantly for **3** and even further for **1**. Isobutyric acid is likewise unable to pass through the pores of **3** or **1**, however due to the missing methyl group of the ligand flexing of a single dimer in the pore unit allows the ligand to reposition such that it can pass through the pore, whereas the symmetry of pivalic acid requires multiple parts of the pore to flex to permit passage. Since the pivalic acid requires much more distortion of the pore to pass through into the interior of the Keplerate it appears that the exchange rate is dominated less by the dimensions of the pore and more by the emergence of an amplified breathability effect as the pore becomes “softer” in the presence of the larger chalcogenide atoms. Selenide is larger, softer, and less electronegative than sulphide. The decreased electronegativity (as evidenced by being more reducing) polarises less electron density towards selenide, which puts more electron density on the Mo^V^, in turn weakening the bond it forms to oxide ligands. The more diffuse orbitals, and occupied d‐orbital character (sulphide possessing no filled d‐orbitals) makes distortion away from the optimal bond geometry a lower cost, greatly assisting bending out of the plane. Bending away from the ideal geometry is partially compensated by d‐orbital bonding that is possible, making the bonds less directional, and thus more flexible. In fact, the selenide already forms a more acute Mo−Se−Mo bond angle, which is further from an ideal geometry, such that distortion is less costly.

Ligand transfer across the pore is thereby controlled by two factors, rigid pore size and pore flexibility, with rigid pore size dominating for “fast” transfer and observed in **3** and **4**, whilst as the ligand size approaches the limit of pore size, the pore flexibility takes over the whole process, with **1** containing more flexible bonds. It is noteworthy that we were unable to find a ligand which could enter **4** but was wholly excluded from **1** despite the dramatic pore size difference, providing more evidence for the importance of increased breathability in **1** facilitating ultimately the transfer of large ligands across the pore.

The observations above indicate that these molecular nano‐containers should show varying selectivity towards smaller ligands. Based on the previous results for single ligand exchange it was predicted that **1** would show less selectivity than **3** and based on the trends in pore flexibility it would then be predicted that **4** would show greater selectivity than either.

### Double ligand exchange

**Figure 5:**
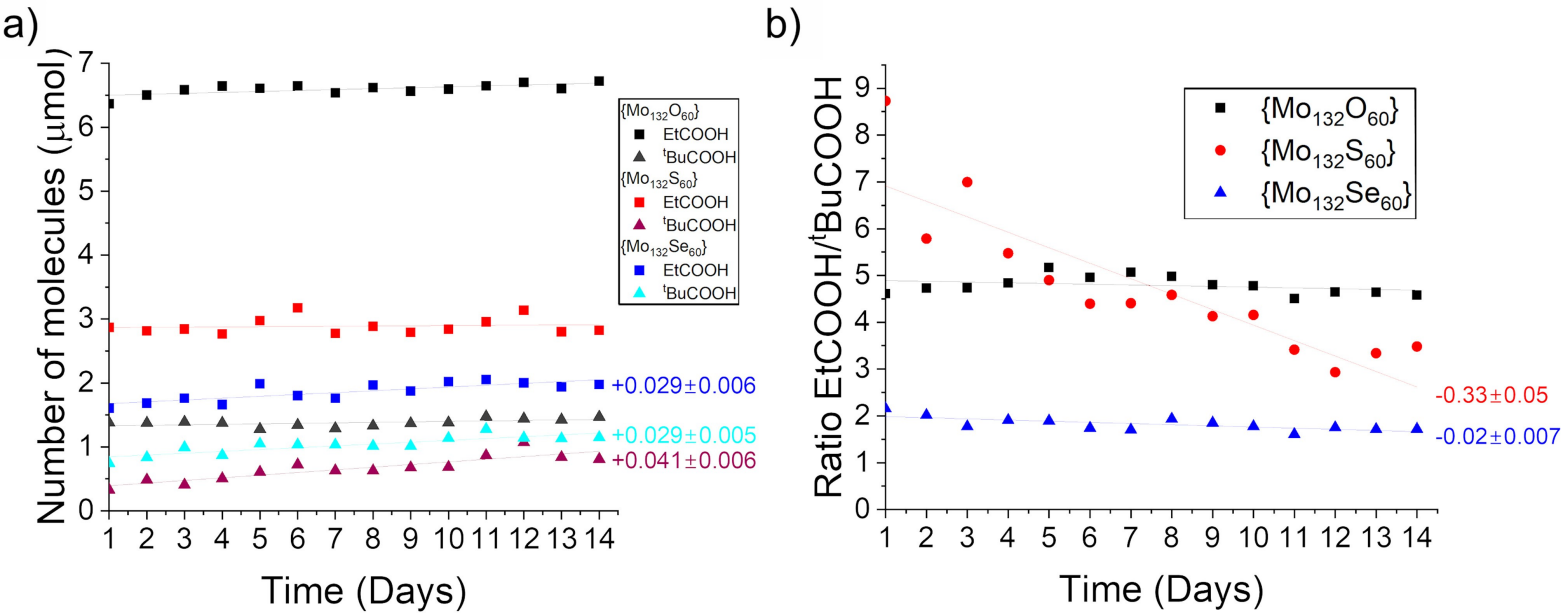
a) Number of internal ligands of Keplerates over time. Black, {Mo_132_O_60_}; Red, {Mo_132_S_60_}; Blue, {Mo_132_Se_60_}; Squares, EtCOOH; Triangles, ^t^BuCOOH. b) Ratios of internal EtCOOH to ^t^BuCOOH of the same Keplerates over time. Black squares, {Mo_132_O_60_}; Red circles, {Mo_132_S_60_}; Blue triangles, {Mo_132_Se_60_}. Linear fit of data is shown as a straight line, the gradient of the line is shown for linear fit with Pearson's R >0.7.

To confirm this hypothesis, we performed ligand exchange in the presence of a mixture of a large and small ligand. Pivalic acid was chosen as the large ligand, however the even smaller propionic acid (R=Et) was used here to improve contrast, whereas in single ligand experiments the exchange was too rapid to be easily resolved. The Keplerate was mixed with a solution containing both ligands at known concentrations and allowed to exchange over the course of 14 days (Figures S5). NMR data was used to estimate the fraction of ligands that had been taken up. Several NMR peaks overlapped. Especially the encapsulated −CH_2_− peaks of propionic acid overlapped strongly with the −CH_3_− peaks from the free ligand, most notably in the selenide cage. With this limitation in mind, we focus here primarily on qualitative conclusions based on comparisons across the three Keplerates. The data are summarized in Figure 5. The exchange within the Keplerates can be compared across the series based on several factors; the total number of each ligand taken up, the rate at which that number changes over the days studied, and the ratio of small ligands to large ligands, which provides an estimate of the selectivity towards small ligands. It should be kept in mind that this investigation occurred over 14 days, and therefore there will be always a balance not only of the kinetic rate of exchange but the ideal position of thermodynamic equilibrium.

In terms of the total number of each ligand taken up, at all stages the number of ligands up taken reflected the relative rates determined previously, with oxygen consistently showing the greatest numbers of both ligands encapsulated, whilst the {Mo_132_S_60_} **3** always contained more propionic ligand than the {Mo_132_Se_60_} **1**, whilst **1** always contained more pivalic ligands than **3**. Comparing the rate of change in this number however deviates from the rates shown in single ligand experiments.

The {Mo_132_S_60_} Keplerate **3** shows no increase in the number of propionate ligands but does show an increase in the number of pivalate ligands, and the {Mo_132_Se_60_} **1** shows an increase of both ligands at an equal rate. This is an inversion of the initial rates observed for single ligand exchange, where smaller ligands exchanged slowest with **1**, and pivalate exchanged slowest with **3**, whereas now the rate is highest for these pairings.

The rate of increase of pivalate ligands is greatest in the S‐Keplerate **3**, however the total number is the least. Since no pivalate ligands were present inside the capsule prior to the experiment this implies that {Mo_132_S_60_} **3** is the furthest from equilibrium, and that rapid, non‐linear exchange occurred within the other two cages prior to the first measurement. The same is true for the exchange of propionate with **1**. The linear rate of increase over the timeframe studied is therefore more so a measure of distance from equilibrium than it is a measure of initial exchange rate, with the number of pivalate ligands inside the {Mo_132_Se_60_} **1** and {Mo_132_O_60_} **4** capsules being closer to their final equilibrium position, hence the lower rate of increase despite an (unobserved) greater rate of increase prior to the first measurement.

Comparing the ratio of propionic acid to pivalic acid describes the degree of selectivity. The {Mo_132_Se_60_} **1** consistently shows the lowest degree of selectivity, although it remains selective at a ratio of about 2 with a minor decrease over the time studied. The {Mo_132_O_60_} **4** shows a ratio of about 5 that remains consistent over the 14 days studied implying that a steady equilibrium had been reached. The {Mo_132_S_60_} **3** however shows a very high selectivity initially of 9, which decreases rapidly, passing the {Mo_132_O_60_} **4** after 5 days and continuing to decrease at a slower rate.

**Figure 6:**
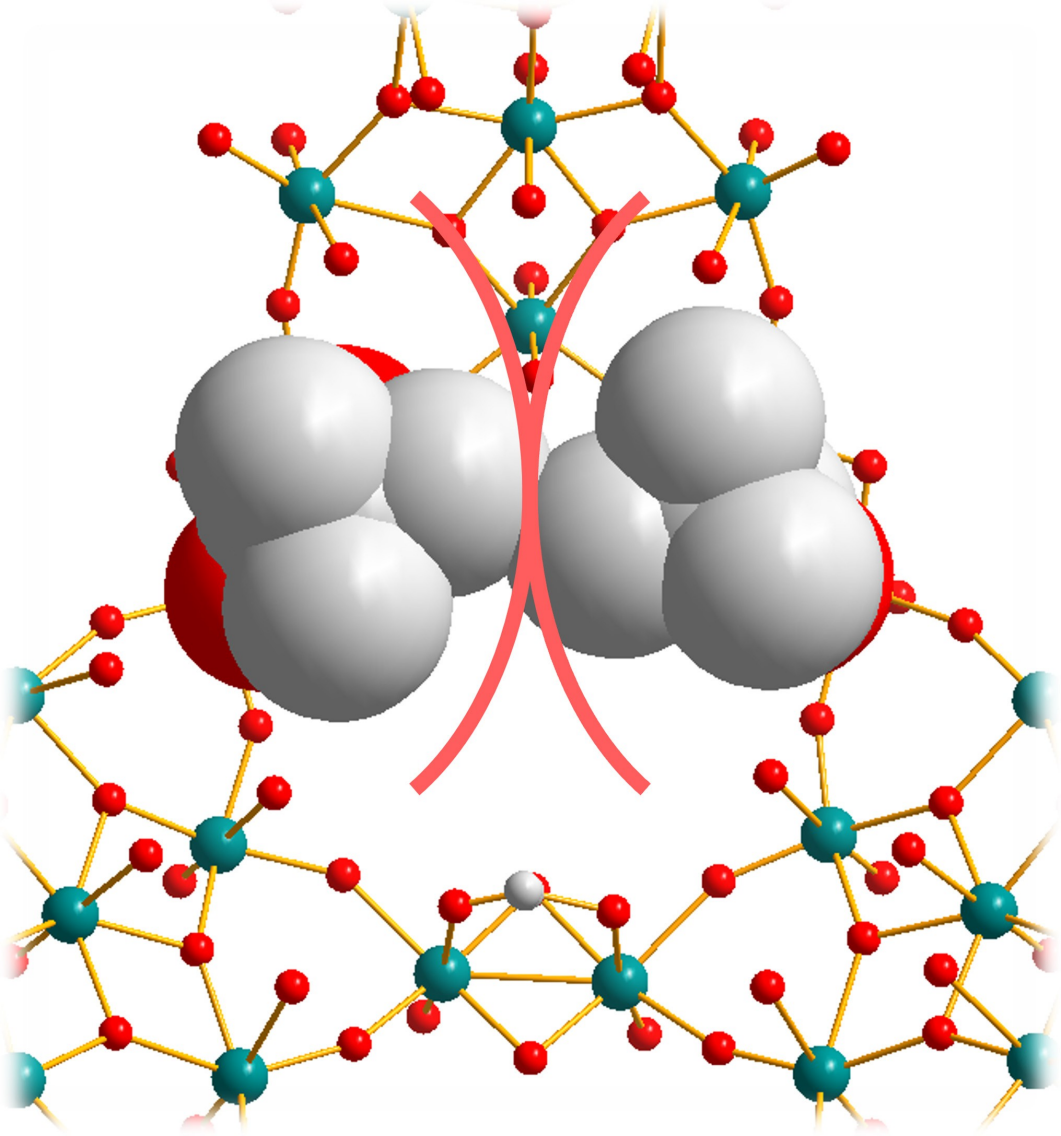
Modelled structure of two pivalic acid ligands occupying adjacent positions within a Keplerate pore, showing steric clash. Ligand show as space filling based on van der Waals radii, other atoms shown as ball‐and‐stick for clarity; Mo, teal; O, red; C white.

The final position of equilibrium is a balance of the entropic force towards mixing, which would tend towards a ratio of 1, against the enthalpic preference for the smaller ligand, largely as a result of steric repulsion from the larger ligand exerted on its neighbours as illustrated in Figure 6. The rate of exchange would accelerate both the entrance and exit of the ligand within the capsule and would therefore not be expected to affect the position of equilibrium. Given the similarity in p*K*_a_ of the two ligands (4.87 for propionic acid, 4.93 for pivalic acid) significant differences in the strength of the bonding would not be expected. A greater preference for ligand attachment would increase the fraction of potential ligand sites occupied, and therefore increase the thermodynamic “cost” of attaching larger ligands, which would in turn move the position of equilibrium to favour small ligands more.

The initial ratio on first mixing however is determined solely by the initial rates of exchange, whilst the overall rate at which equilibrium is reached is controlled by the slowest rate of exchange (e.g. pivalic acid). That the sulphide capsule shows the greatest change in ratio can therefore be rationalised as a consequence of slow pivalic acid exchange meaning that equilibrium is approached slower. Since the ratio decreases in order to reach equilibrium, it is clear that the selectivity based on kinetic rate of exchange is greater than the selectivity based on position of equilibrium. In this way it is the {Mo_132_S_60_} **3** capsule that maintains a high degree of selectivity the longest, however its final selectivity (over prolonged time) is less than the {Mo_132_O_60_} **4**. This creates a scenario in which the {Mo_132_O_60_} **4** is simultaneously the most selective under full kinetic control and full thermodynamic control, but because the {Mo_132_S_60_} **3** maintains kinetic control for longer there is a brief window in which it shows higher selectivity.

The fact that {Mo_132_O_60_} **4** shows the highest selectivity at equilibrium implies it shows the strongest preference for ligand attachment. A simple argument in terms of decreasing electronegativity descending the series O>S>Se would imply a stronger positive charge on Mo^V^ in the {Mo_132_O_60_} **4**, which in turn would therefore show a stronger preference for the negatively charged incoming ligand. The difference in electronegativity of sulphur and selenium is smaller, however it is still large enough to manifest as a 0.1 V difference in the standard reduction potential of the E/E^2−^ couple.^[24]^ It is further observed that structures containing the [Mo^V^_2_(μ‐O)_2_O_2_]^2+^ motif are invariably supported by negatively charged ligands[^1^, ^25^, ^26^, ^27^, ^28^, ^29^] coordinated to the axial position, whilst the [Mo^V^_2_(μ‐S)_2_O_2_]^2+^ and [Mo^V^_2_(μ‐Se)_2_O_2_]^2+^ are found supported by water[^30^, ^31^] on their axial positions, or with uncoordinated axial positions.[^32^, ^33^, ^34^]

### Ligand extraction

**Figure 7:**
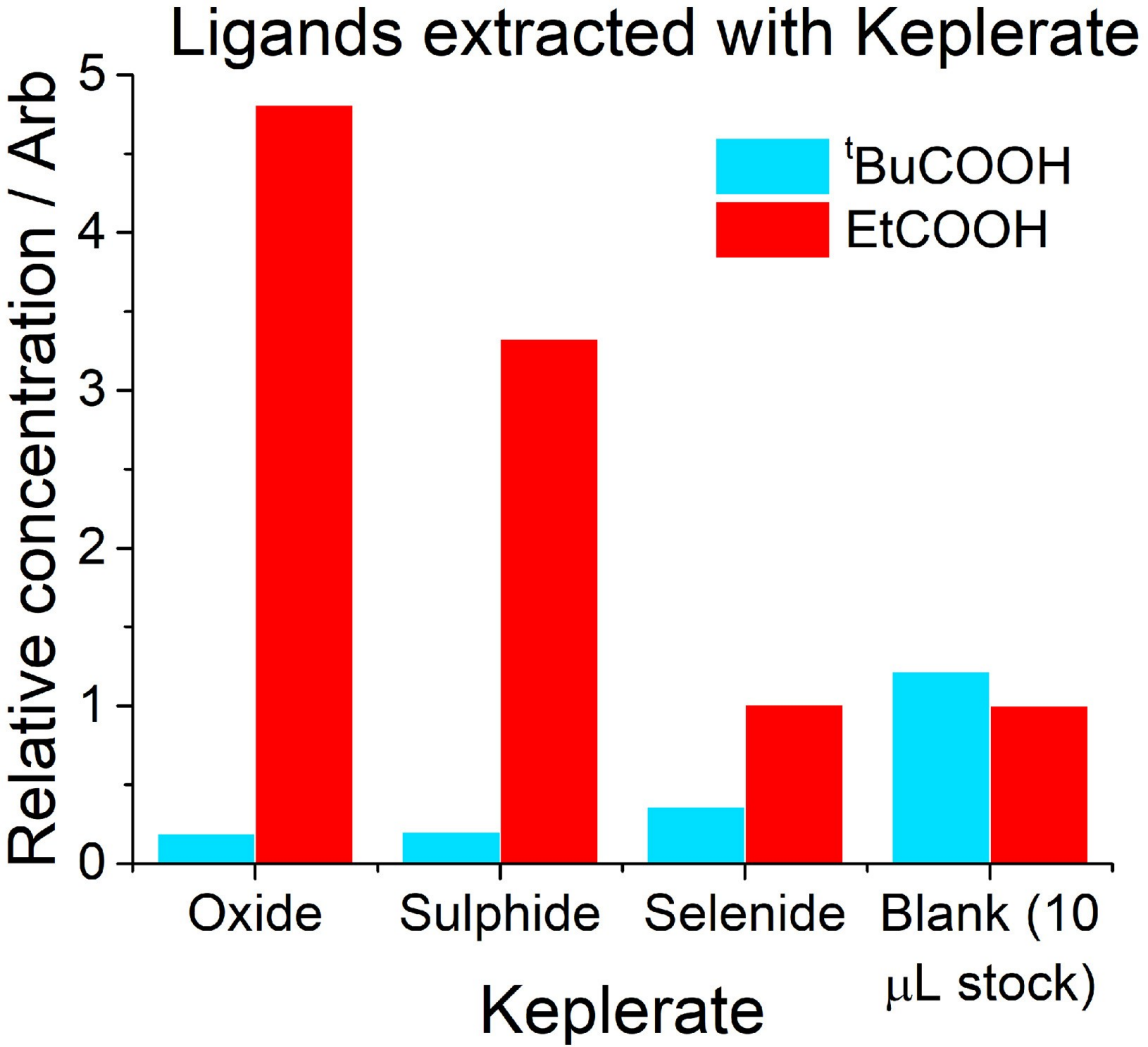
Relative concentrations of solutions of ligands extracted using {Mo_132_O_60_} **4**, {Mo_132_S_60_} **3** and {Mo_132_Se_60_} **1**, Keplerates, alongside the concentrations present in stock solution of ligands before extraction.

To further explore the capacity of these Keplerates to selectively extract small molecules from solution based on size we exploited the Keplerates high hydrophilicity to rapidly precipitate them from solution by adding an excess of ethanol (Figures 7 and S6). The Keplerates separated this way would contain a “snapshot” of the current contents of the capsule, which could be extracted by the breaking of the capsule with addition of base, thereby demonstrating the ability of the Keplerates to function as a molecular sieve and extract ligands of a certain size from solution with a high degree of selectivity. As a secondary benefit doing so would greatly sharpen the NMR spectra compared to encapsulated ligands, which allows for more reliable quantitative data. Both ligands used were highly soluble in or miscible with ethanol, and thus were not expected to precipitate in their free forms. Since the Keplerates showed greater kinetic selectivity than thermodynamic selectivity, mixing of ligand occurred over 30 minutes, as a balance of allowing enough ligands to enter the capsule, whilst simultaneously minimising the equilibration of the two ligands inside the capsule.

The found ratios showed considerably greater selectivity in each case than the mixtures which were allowed to equilibrate, indicating that the selectivity here is kinetically controlled. In each case propionic acid was in excess, with excess of 92.4 % found in **4**, 88.7 % for **3** and 47.3 % in **1**.

In line with the single ligand measurements, the rate of propionic acid uptake follows pore size O>S>Se very clearly. This is to be expected, with selenide showing by a considerable margin the least uptake. The uptake of pivalic acid however shows the reverse pattern Se>S>O. This implies two things. Firstly, the selectivity of {Mo_132_O_60_} is, as theorised, higher than {Mo_132_Se_60_} or {Mo_132_S_60_}. However, despite this, based on single ligand experiments it would still be expected to show the most uptake with either ligand. Instead, it shows the least uptake of pivalic acid (but the highest total uptake). This may be rationalised by invoking a competitive effect between the two ligands (Figure 6), in agreement with previous observations. Both are less acidic than acetic acid, with a p*K*_a_ difference of over 0.1 (acetic acid p*K*_a_ 4.75 compared to 4.87 for propionic acid and 4.93 for pivalic acid), and thus will be less labile once coordinated. Further both provide greater steric competition, especially pivalic acid, however the steric effect pivalic acid exerts will be greater on propionic than on acetic acid. Therefore, rapid occupation of sites by propionic acid over the thirty minutes would attenuate the attachment of pivalic acid via competition, both through reducing the availability of sites, and through steric repulsion towards adjacent sites, leading to an overall lower uptake than in other Keplerates, despite more rapid transport across the pores. This competition would be present for other Keplerates as the concentration of propionic acid built up as well, and to show lower pivalic acid uptake than other Keplerates, as was observed, the relative rate decreases on going from the small propionic acid to the large pivalic acid must be higher, and therefore more selective. This trend continues, with {Mo_132_Se_60_} showing both the least propionic acid uptake, but a considerable increase in pivalic uptake compared to the other Keplerates. It does still show a moderate degree of selectivity, as approximately three times as much propionic acid was extracted, however the simultaneous shrinking of the pore, alongside an increase in pore flexibility moves towards a pore that is size agnostic.

The total number of propanoate ligands extracted on the other hand closely follows the observed rates for single ligand experiments. Since the majority of the coordination sites are occupied by propanoate the behaviour would be expected to be much closer to the behaviour in the presence of only a single ligand.

## Conclusion

Two new additions to the Keplerate family of elusive structures using [Mo_2_O_2_Se_2_]^2+^ as a linker has been synthesised with both molybdate and tungstate pentagonal units giving an {Mo_132_Se_60_} **1** and {W_72_Mo_60_Se_60_} **2** type capsules, respectively. These new members have very similar structural features to the previously synthesised {Mo_132_}, {Mo_132_S_60_}, {W_72_Mo_60_} and {W_72_Mo_60_S_60_} featuring internal acetate ligands attached to the dimeric linker units. However, compared to the other structures the hexagonal Se_3_O_6_ pores are even more contracted compared to S_3_O_6_ pores. This provides a range of pore radii from 1.78 Å in {Mo_132_O_60_} Keplerate **4**, to 0.94 Å in the new {Mo_132_Se_60_} Keplerate **1** without modifying any other parts of the Keplerate topology. The engineering of appropriate pore size could be exploited to control the exchange of small molecules across the pore of the molecular nano‐containers. Interestingly, it was found that the largest molecules travelled most slowly through the pores of the {Mo_132_S_60_} Keplerate **3**, despite being wider in rigid pore size than ones in the {Mo_132_Se_60_} Keplerate **1**. This unexpected emergence of increased pore breathability further highlights the importance of multiple parameters that facilitate mass transport processes in supramolecular systems.

The overall selectivity of Keplerates, particularly the {Mo_132_O_60_} **4**, for small ligands such as propionic acid instead of larger ligands such as pivalic acid, is very high at an excess of 92 % after 30 minutes of stirring which is indicative of its wide dimensions and rigidity of its pores. The selectivity is believed to arise simultaneously from kinetic preference for small molecules crossing the pores, and thermodynamic preference to avoid steric clash from larger ligands, however far greater selectivity is observed under kinetic control. The profile of relative selectivity varies, with the {Mo_132_O_60_} **4** and the {Mo_132_S_60_} **3** presenting the highest selectivity at different timeframes.

In marked contrast, the selectivity of the {Mo_132_Se_60_} **1** was consistently low across all timeframes, showing only a minor preference for the smaller ligands. This size‐agnostic behaviour is believed to arise from the increased flexibility of the Mo−Se bonds coupled with the small pore aperture necessitating pore flexing even for small molecules.

Better control of pore size can give control over the transport of small molecules across Keplerates without completely blocking the pores allowing larger molecules to be excluded or sufficiently slowed in crossing the pore, with different pore sizes that can be tuned for specific applications. It becomes evident that the kinetics or the thermodynamics of the system can be used for the effective extraction of organic content by restricting the exchange process at the specific timescale of interest in aqueous media within a range of mild pH. This offers prospective applications in drug delivery systems, selective molecular sieving, catalysis within confined spaces and sensing.

## Conflict of interest

The authors declare no conflict of interest.

1

## Supporting information

As a service to our authors and readers, this journal provides supporting information supplied by the authors. Such materials are peer reviewed and may be re‐organized for online delivery, but are not copy‐edited or typeset. Technical support issues arising from supporting information (other than missing files) should be addressed to the authors.

Supporting Information

Supporting Information

Supporting Information

Supporting Information

Supporting Information

## Data Availability

The data that support the findings of this study are available in the Supporting Information of this article.
