# Supplementary material for: Pore “Softening” and Emergence of Breathability Effects of New Keplerate Nano‐Containers
Source: Angew Chem Int Ed Engl. 2023 Mar 9;62(20):e202218897. doi: 10.1002/anie.202218897 (PMC10946700; doi:10.1002/anie.202218897)

checkCIF/PLATON report

Structure factors have been supplied for datablock(s) ae432e_sq

THIS REPORT IS FOR GUIDANCE ONLY. IF USED AS PART OF A REVIEW PROCEDURE FOR PUBLICATION, IT SHOULD NOT REPLACE THE EXPERTISE OF AN EXPERIENCED CRYSTALLOGRAPHIC REFEREE.

No syntax errors found. CIF dictionary Interpreting this report

Datablock: ae432e_sq

```
Bond precision:    = 0.0000 A              Wavelength=0.71073

Cell:              a=46.3086(3)      b=46.3086(3)      c=46.3086(3)
                   alpha=90          beta=90           gamma=90
Temperature:       150 K
```

	Calculated	Reported
Volume	99308.2(19)	99308.2(19)
Space group	F m -3 m	F m -3 m
Hall group	-F 4 2 3	-F 4 2 3
Moiety formula	C48 Mo264 O888 Se120, 76.8(O), 12(C) [+ solvent]	?
Sum formula	C60 Mo264 O964.80 Se120 [+ solvent]	C60 H802 Mo132 N42 O644 Se60
Mr	50960.75	29823.07
Dx,g cm-3	1.704	1.995
Z	2	4
Mu (mm-1)	3.861	3.889
F000	46492.8	56768.0
F000'	45584.51	
h,k,lmax	54,54,54	54,54,54
Nref	4121	4106
Tmin,Tmax	0.692,0.774	0.600,1.000
Tmin'	0.226	

```
Correction method= # Reported T Limits: Tmin=0.600 Tmax=1.000
AbsCorr = GAUSSIAN

Data completeness= 0.996           Theta(max)= 24.726
```

R(reflections)= 0.0775(2753)

wR2(reflections)= 0.2832(4106)

S = 1.056

Npar= 255

The following ALERTS were generated. Each ALERT has the format
test-name_ALERT_alert-type_alert-level.
Click on the hyperlinks for more details of the test.

Alert level B

```
PLAT241_ALERT_2_B High   'MainMol' Ueq as Compared to Neighbors of        O14 Check
PLAT241_ALERT_2_B High   'MainMol' Ueq as Compared to Neighbors of        O16 Check
PLAT306_ALERT_2_B Isolated Oxygen Atom (H-atoms Missing ?) .......        O20 Check
```

Alert level C

```
THETM01_ALERT_3_C  The value of sine(theta_max)/wavelength is less than 0.590
            Calculated sin(theta_max)/wavelength =    0.5885
PLAT084_ALERT_3_C High wR2 Value (i.e. > 0.25) ...................       0.28 Report
PLAT202_ALERT_3_C Isotropic non-H Atoms in Anion/Solvent .........          2 Check
              O20     O22
PLAT241_ALERT_2_C High   'MainMol' Ueq as Compared to Neighbors of        O13 Check
PLAT241_ALERT_2_C High   'MainMol' Ueq as Compared to Neighbors of        O15 Check
PLAT260_ALERT_2_C Large Average Ueq of Residue Including       Mo1      0.108 Check
PLAT905_ALERT_3_C Negative K value in the Analysis of Variance ...     -2.354 Report
PLAT910_ALERT_3_C Missing # of FCF Reflection(s) Below Theta(Min).          9 Note
PLAT911_ALERT_3_C Missing FCF Refl Between Thmin & STh/L=    0.589          3 Report
PLAT918_ALERT_3_C Reflection(s) with I(obs) much Smaller I(calc) .          3 Check
PLAT971_ALERT_2_C Check Calcd Resid. Dens.  0.07Ang From C1              1.54 eA-3
PLAT975_ALERT_2_C Check Calcd Resid. Dens.  0.49Ang From O8       .      0.68 eA-3
PLAT975_ALERT_2_C Check Calcd Resid. Dens.  0.56Ang From O22      .      0.58 eA-3
PLAT975_ALERT_2_C Check Calcd Resid. Dens.  0.64Ang From O23      .      0.45 eA-3
PLAT975_ALERT_2_C Check Calcd Resid. Dens.  0.71Ang From O13      .      0.44 eA-3
PLAT976_ALERT_2_C Check Calcd Resid. Dens.  0.45Ang From O16      .     -0.90 eA-3
PLAT976_ALERT_2_C Check Calcd Resid. Dens.  0.76Ang From O20      .     -0.45 eA-3
```

Alert level G

```
FORMU01_ALERT_2_G  There is a discrepancy between the atom counts in the
            _chemical_formula_sum and the formula from the _atom_site* data.
            Atom count from _chemical_formula_sum:C60 H802 Mo132 N42 O644 Se60
            Atom count from the _atom_site data:  C30 Mo132 O482.4 Se60
CELLZ01_ALERT_1_G Difference between formula and atom_site contents detected.
CELLZ01_ALERT_1_G ALERT: Large difference may be due to a
            symmetry error - see SYMMG tests
           From the CIF: _cell_formula_units_Z    4
           From the CIF: _chemical_formula_sum  C60 H802 Mo132 N42 O644 Se60
           TEST: Compare cell contents of formula and atom_site data
```

atom	Z*formula	cif sites	diff
C	240.00	120.00	120.00
H	3208.00	0.00	3208.00
Mo	528.00	528.00	0.00
N	168.00	0.00	168.00
O	2576.00	1929.60	646.40

```
          Se       240.00    240.00    0.00
PLAT002_ALERT_2_G Number of Distance or Angle Restraints on AtSite          7 Note
PLAT003_ALERT_2_G Number of Uiso or Uij Restrained non-H Atoms ...         25 Report
PLAT040_ALERT_1_G No H-atoms in this Carbon Containing Compound ..     Please Check
PLAT041_ALERT_1_G Calc. and Reported SumFormula    Strings  Differ     Please Check
PLAT045_ALERT_1_G Calculated and Reported Z Differ by a Factor ...      0.500 Check
PLAT083_ALERT_2_G SHELXL Second Parameter in WGHT  Unusually Large    1558.61 Why ?
PLAT172_ALERT_4_G The CIF-Embedded .res File Contains DFIX Records          3 Report
PLAT178_ALERT_4_G The CIF-Embedded .res File Contains SIMU Records          2 Report
PLAT186_ALERT_4_G The CIF-Embedded .res File Contains ISOR Records          2 Report
PLAT188_ALERT_3_G A Non-default SIMU Restraint Value has been used     0.0100 Report
PLAT188_ALERT_3_G A Non-default SIMU Restraint Value has been used     0.0100 Report
PLAT300_ALERT_4_G Atom Site Occupancy of Mo1        Constrained at        0.5 Check
PLAT300_ALERT_4_G Atom Site Occupancy of Mo2        Constrained at        0.5 Check
PLAT300_ALERT_4_G Atom Site Occupancy of Mo3        Constrained at       0.55 Check
PLAT300_ALERT_4_G Atom Site Occupancy of Mo5        Constrained at        0.5 Check
PLAT300_ALERT_4_G Atom Site Occupancy of Mo6        Constrained at       0.62 Check
PLAT300_ALERT_4_G Atom Site Occupancy of Mo3'       Constrained at       0.45 Check
PLAT300_ALERT_4_G Atom Site Occupancy of Mo4        Constrained at       0.35 Check
PLAT300_ALERT_4_G Atom Site Occupancy of Mo4'       Constrained at       0.15 Check
PLAT300_ALERT_4_G Atom Site Occupancy of Mo6'       Constrained at       0.38 Check
PLAT300_ALERT_4_G Atom Site Occupancy of Se1        Constrained at        0.5 Check
PLAT300_ALERT_4_G Atom Site Occupancy of Se2        Constrained at        0.5 Check
PLAT300_ALERT_4_G Atom Site Occupancy of Se3        Constrained at        0.5 Check
PLAT300_ALERT_4_G Atom Site Occupancy of O1         Constrained at        0.5 Check
PLAT300_ALERT_4_G Atom Site Occupancy of O2         Constrained at        0.5 Check
PLAT300_ALERT_4_G Atom Site Occupancy of O3         Constrained at        0.5 Check
PLAT300_ALERT_4_G Atom Site Occupancy of O4         Constrained at        0.5 Check
PLAT300_ALERT_4_G Atom Site Occupancy of O5         Constrained at        0.5 Check
PLAT300_ALERT_4_G Atom Site Occupancy of O6         Constrained at        0.5 Check
PLAT300_ALERT_4_G Atom Site Occupancy of O7         Constrained at        0.5 Check
PLAT300_ALERT_4_G Atom Site Occupancy of O8         Constrained at        0.5 Check
PLAT300_ALERT_4_G Atom Site Occupancy of O9         Constrained at        0.5 Check
PLAT300_ALERT_4_G Atom Site Occupancy of O11        Constrained at        0.5 Check
PLAT300_ALERT_4_G Atom Site Occupancy of O18        Constrained at        0.5 Check
PLAT300_ALERT_4_G Atom Site Occupancy of O19        Constrained at        0.5 Check
PLAT300_ALERT_4_G Atom Site Occupancy of C2         Constrained at        0.5 Check
PLAT300_ALERT_4_G Atom Site Occupancy of O22        Constrained at        0.6 Check
PLAT300_ALERT_4_G Atom Site Occupancy of O21        Constrained at        0.4 Check
PLAT300_ALERT_4_G Atom Site Occupancy of O21'       Constrained at       0.24 Check
PLAT300_ALERT_4_G Atom Site Occupancy of O23        Constrained at       0.16 Check
PLAT301_ALERT_3_G Main Residue  Disorder .............(Resd  1  )        69% Note
PLAT302_ALERT_4_G Anion/Solvent/Minor-Residue Disorder (Resd  3  )      100% Note
PLAT302_ALERT_4_G Anion/Solvent/Minor-Residue Disorder (Resd  4  )      100% Note
PLAT302_ALERT_4_G Anion/Solvent/Minor-Residue Disorder (Resd  5  )      100% Note
PLAT302_ALERT_4_G Anion/Solvent/Minor-Residue Disorder (Resd  6  )      100% Note
PLAT304_ALERT_4_G Non-Integer Number of Atoms in ..... (Resd  1  )     137.50 Check
PLAT304_ALERT_4_G Non-Integer Number of Atoms in ..... (Resd  2  )       0.25 Check
PLAT304_ALERT_4_G Non-Integer Number of Atoms in ..... (Resd  3  )       0.15 Check
PLAT304_ALERT_4_G Non-Integer Number of Atoms in ..... (Resd  4  )       0.20 Check
PLAT304_ALERT_4_G Non-Integer Number of Atoms in ..... (Resd  5  )       0.12 Check
PLAT304_ALERT_4_G Non-Integer Number of Atoms in ..... (Resd  6  )       0.08 Check
PLAT304_ALERT_4_G Non-Integer Number of Atoms in ..... (Resd  7  )       0.12 Check
PLAT311_ALERT_2_G Isolated Disordered Oxygen Atom (No H's ?) .....         O4 Check
PLAT311_ALERT_2_G Isolated Disordered Oxygen Atom (No H's ?) .....         O5 Check
PLAT311_ALERT_2_G Isolated Disordered Oxygen Atom (No H's ?) .....        O22 Check
PLAT311_ALERT_2_G Isolated Disordered Oxygen Atom (No H's ?) .....        O21 Check
```

```
PLAT311_ALERT_2_G Isolated Disordered Oxygen Atom (No H's ?) .....       O21' Check
PLAT311_ALERT_2_G Isolated Disordered Oxygen Atom (No H's ?) .....        O23 Check
PLAT606_ALERT_4_G Solvent Accessible VOID(S) in Structure .......           ! Info
PLAT811_ALERT_5_G No ADDSYM Analysis: Too Many Excluded Atoms ....          ! Info
PLAT860_ALERT_3_G Number of Least-Squares Restraints ............         142 Note
PLAT869_ALERT_4_G ALERTS Related to the Use of SQUEEZE  Suppressed          ! Info
PLAT909_ALERT_3_G Percentage of I>2sig(I) Data at Theta(Max) Still        31% Note
PLAT913_ALERT_3_G Missing # of Very Strong Reflections in FCF ....          1 Note
PLAT933_ALERT_2_G Number of HKL-OMIT Records in Embedded .res File          2 Note
PLAT960_ALERT_3_G Number of Intensities with I  <   - 2*sig(I) ...          7 Check
PLAT965_ALERT_2_G The SHELXL WEIGHT Optimisation has not Converged     Please Check
PLAT967_ALERT_5_G Note: Two-Theta Cutoff Value in Embedded .res ..       52.0 Degree
```

```
  0 ALERT level A = Most likely a serious problem - resolve or explain
  3 ALERT level B = A potentially serious problem, consider carefully
 17 ALERT level C = Check. Ensure it is not caused by an omission or oversight
 71 ALERT level G = General information/check it is not something unexpected

  5 ALERT type 1 CIF construction/syntax error, inconsistent or missing data
 25 ALERT type 2 Indicator that the structure model may be wrong or deficient
 14 ALERT type 3 Indicator that the structure quality may be low
 45 ALERT type 4 Improvement, methodology, query or suggestion
  2 ALERT type 5 Informative message, check
```

It is advisable to attempt to resolve as many as possible of the alerts in all categories. Often the minor alerts point to easily fixed oversights, errors and omissions in your CIF or refinement strategy, so attention to these fine details can be worthwhile. In order to resolve some of the more serious problems it may be necessary to carry out additional measurements or structure refinements. However, the purpose of your study may justify the reported deviations and the more serious of these should normally be commented upon in the discussion or experimental section of a paper or in the "special_details" fields of the CIF. checkCIF was carefully designed to identify outliers and unusual parameters, but every test has its limitations and alerts that are not important in a particular case may appear. Conversely, the absence of alerts does not guarantee there are no aspects of the results needing attention. It is up to the individual to critically assess their own results and, if necessary, seek expert advice.

Publication of your CIF in IUCr journals

A basic structural check has been run on your CIF. These basic checks will be run on all CIFs submitted for publication in IUCr journals (*Acta Crystallographica*, *Journal of Applied Crystallography*, *Journal of Synchrotron Radiation*); however, if you intend to submit to *Acta Crystallographica Section C* or *E* or *IUCrData*, you should make sure that full publication checks are run on the final version of your CIF prior to submission.

Publication of your CIF in other journals

Please refer to the *Notes for Authors* of the relevant journal for any special instructions relating to CIF submission.

PLATON version of 28/11/2022; check.def file version of 28/11/2022

Datablock ae432e_sq - ellipsoid plot

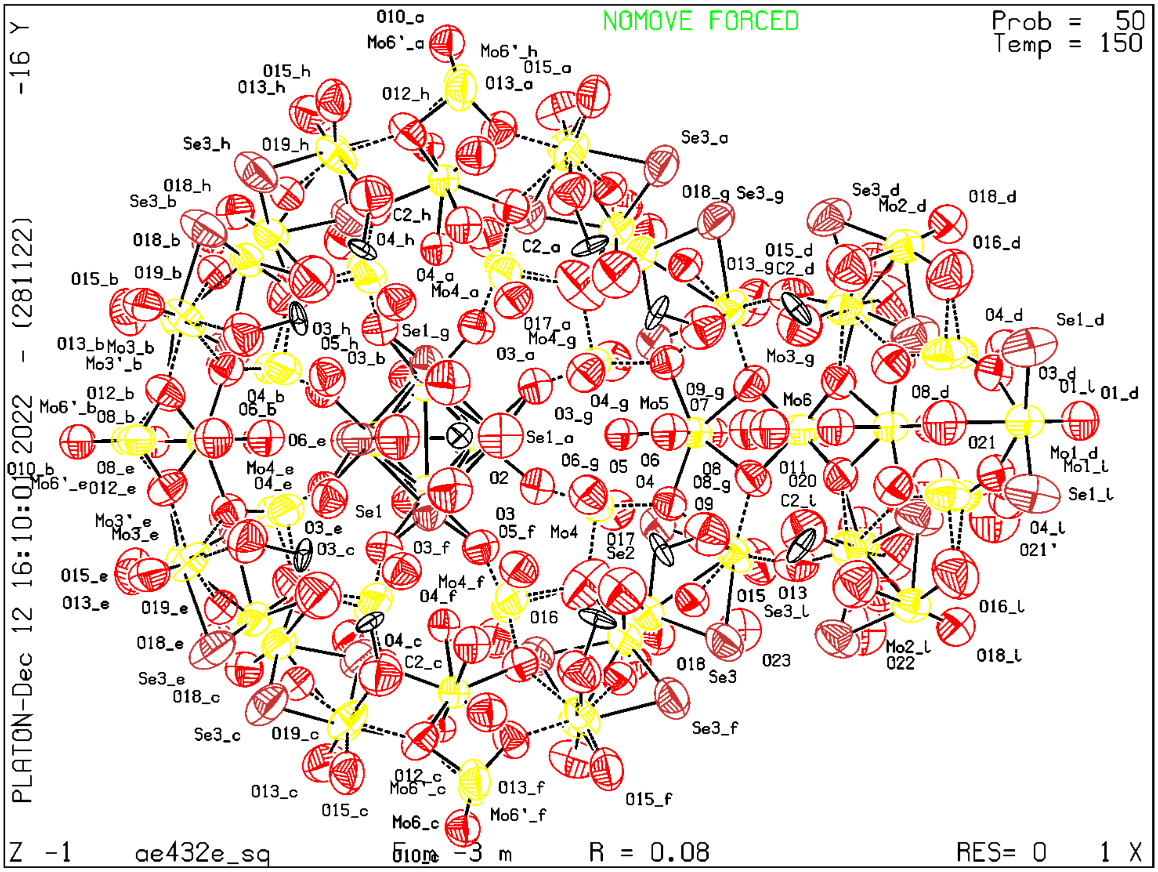

Supplement: Supplementary file 2 — Supporting Information [file ANIE-62-0-s005.pdf]
