# Supplementary material for: Pore “Softening” and Emergence of Breathability Effects of New Keplerate Nano‐Containers
Source: Angew Chem Int Ed Engl. 2023 Mar 9;62(20):e202218897. doi: 10.1002/anie.202218897 (PMC10946700; doi:10.1002/anie.202218897)

checkCIF/PLATON report

Structure factors have been supplied for datablock(s) ae448i_sq

THIS REPORT IS FOR GUIDANCE ONLY. IF USED AS PART OF A REVIEW PROCEDURE FOR PUBLICATION, IT SHOULD NOT REPLACE THE EXPERTISE OF AN EXPERIENCED CRYSTALLOGRAPHIC REFEREE.

No syntax errors found. CIF dictionary Interpreting this report

Datablock: ae448i_sq

Bond precision:	= 0.0000 A		Wavelength=0.71073
Cell:	a=46.7155(4)	b=46.7155(4)	c=46.7155(4)
	alpha=90	beta=90	gamma=90
Temperature:	150 K		

	Calculated	Reported
Volume	101949(3)	101949(3)
Space group	F m -3 m	F m -3 m
Hall group	-F 4 2 3	-F 4 2 3
Moiety formula	C90 Mo120 O888 Se96 W144, 12(Se2), 96(O0.23), ***(O) [+ solvent	?
Sum formula	C90 Mo120 O971.52 Se120 W144 [+ solvent]	C60 H802 Mo60 N42 O644 Se60 W72
Mr	64086.18	36152.59
Dx,g cm-3	2.088	2.355
Z	2	4
Mu (mm-1)	10.994	11.020
F000	56176.3	65984.0
F000'	55563.45	
h,k,lmax	54,54,54	54,54,52
Nref	4216	4207
Tmin,Tmax	0.046,0.516	0.339,0.531
Tmin'	0.023	

Correction method= # Reported T Limits: Tmin=0.339 Tmax=0.531
AbsCorr = GAUSSIAN

Data completeness= 0.998 Theta(max)= 24.728

R(reflections)= 0.0557(2632)   wR2(reflections)= 0.1956(4207)

S = 1.025   Npar= 274

The following ALERTS were generated. Each ALERT has the format
test-name_ALERT_alert-type_alert-level.
Click on the hyperlinks for more details of the test.

Alert level B

```
PLAT201_ALERT_2_B Isotropic non-H Atoms in Main Residue(s) .......          2 Report
              O15'      C1
PLAT241_ALERT_2_B High   'MainMol' Ueq as Compared to Neighbors of        O13 Check
PLAT241_ALERT_2_B High   'MainMol' Ueq as Compared to Neighbors of        O17 Check
```

Alert level C

```
THETM01_ALERT_3_C  The value of sine(theta_max)/wavelength is less than 0.590
            Calculated sin(theta_max)/wavelength =    0.5886
PLAT241_ALERT_2_C High   'MainMol' Ueq as Compared to Neighbors of        O14 Check
PLAT260_ALERT_2_C Large Average Ueq of Residue Including        W1      0.106 Check
PLAT905_ALERT_3_C Negative K value in the Analysis of Variance ...     -3.517 Report
PLAT905_ALERT_3_C Negative K value in the Analysis of Variance ...     -0.199 Report
PLAT910_ALERT_3_C Missing # of FCF Reflection(s) Below Theta(Min).          9 Note
PLAT911_ALERT_3_C Missing FCF Refl Between Thmin & STh/L=    0.589          2 Report
PLAT918_ALERT_3_C Reflection(s) with I(obs) much Smaller I(calc) .          1 Check
PLAT973_ALERT_2_C Check Calcd Positive Resid. Density on        W3       1.23 eA-3
PLAT975_ALERT_2_C Check Calcd Resid. Dens.  0.89Ang From O22"    .       1.00 eA-3
PLAT975_ALERT_2_C Check Calcd Resid. Dens.  0.54Ang From O24'    .       0.60 eA-3
PLAT975_ALERT_2_C Check Calcd Resid. Dens.  0.85Ang From O7      .       0.55 eA-3
PLAT975_ALERT_2_C Check Calcd Resid. Dens.  0.93Ang From O22     .       0.52 eA-3
PLAT975_ALERT_2_C Check Calcd Resid. Dens.  0.84Ang From O5      .       0.52 eA-3
PLAT976_ALERT_2_C Check Calcd Resid. Dens.  0.67Ang From O16     .      -0.75 eA-3
```

Alert level G

```
FORMU01_ALERT_2_G  There is a discrepancy between the atom counts in the
            _chemical_formula_sum and the formula from the _atom_site* data.
            Atom count from _chemical_formula_sum:C60 H802 Mo60 N42 O644 Se60 W72
            Atom count from the _atom_site data:  C45 Mo60 O485.7600 Se60 W72
CELLZ01_ALERT_1_G Difference between formula and atom_site contents detected.
CELLZ01_ALERT_1_G ALERT: Large difference may be due to a
            symmetry error - see SYMMG tests
           From the CIF: _cell_formula_units_Z    4
           From the CIF: _chemical_formula_sum  C60 H802 Mo60 N42 O644 Se60 W72
           TEST: Compare cell contents of formula and atom_site data
```

atom	Z*formula	cif sites	diff
C	240.00	180.00	60.00
H	3208.00	0.00	3208.00
Mo	240.00	240.00	0.00
N	168.00	0.00	168.00
O	2576.00	1943.04	632.96
Se	240.00	240.00	0.00
W	288.00	288.00	0.00

```
PLAT002_ALERT_2_G Number of Distance or Angle Restraints on AtSite         14 Note
PLAT003_ALERT_2_G Number of Uiso or Uij Restrained non-H Atoms ...         26 Report
PLAT040_ALERT_1_G No H-atoms in this Carbon Containing Compound ..     Please Check
PLAT041_ALERT_1_G Calc. and Reported SumFormula    Strings  Differ     Please Check
PLAT045_ALERT_1_G Calculated and Reported Z Differ by a Factor ...      0.500 Check
PLAT083_ALERT_2_G SHELXL Second Parameter in WGHT  Unusually Large    3188.33 Why ?
PLAT172_ALERT_4_G The CIF-Embedded .res File Contains DFIX Records          6 Report
PLAT176_ALERT_4_G The CIF-Embedded .res File Contains SADI Records          2 Report
PLAT178_ALERT_4_G The CIF-Embedded .res File Contains SIMU Records          5 Report
PLAT186_ALERT_4_G The CIF-Embedded .res File Contains ISOR Records          2 Report
PLAT188_ALERT_3_G A Non-default SIMU Restraint Value has been used     0.0100 Report
PLAT188_ALERT_3_G A Non-default SIMU Restraint Value has been used     0.0100 Report
PLAT188_ALERT_3_G A Non-default SIMU Restraint Value has been used     0.0100 Report
PLAT188_ALERT_3_G A Non-default SIMU Restraint Value has been used     0.0100 Report
PLAT188_ALERT_3_G A Non-default SIMU Restraint Value has been used     0.0100 Report
PLAT191_ALERT_3_G A Non-default SADI Restraint Value has been used     0.0100 Report
PLAT191_ALERT_3_G A Non-default SADI Restraint Value has been used     0.0100 Report
PLAT300_ALERT_4_G Atom Site Occupancy of W1         Constrained at        0.5 Check
PLAT300_ALERT_4_G Atom Site Occupancy of W3         Constrained at        0.5 Check
PLAT300_ALERT_4_G Atom Site Occupancy of Mo1        Constrained at        0.5 Check
PLAT300_ALERT_4_G Atom Site Occupancy of Mo2        Constrained at        0.5 Check
PLAT300_ALERT_4_G Atom Site Occupancy of Mo3        Constrained at        0.5 Check
PLAT300_ALERT_4_G Atom Site Occupancy of Se1        Constrained at        0.5 Check
PLAT300_ALERT_4_G Atom Site Occupancy of Se2        Constrained at        0.5 Check
PLAT300_ALERT_4_G Atom Site Occupancy of Se3        Constrained at       0.25 Check
PLAT300_ALERT_4_G Atom Site Occupancy of O1         Constrained at        0.5 Check
PLAT300_ALERT_4_G Atom Site Occupancy of O2         Constrained at        0.5 Check
PLAT300_ALERT_4_G Atom Site Occupancy of O3         Constrained at        0.5 Check
PLAT300_ALERT_4_G Atom Site Occupancy of O4         Constrained at        0.5 Check
PLAT300_ALERT_4_G Atom Site Occupancy of O5         Constrained at        0.5 Check
PLAT300_ALERT_4_G Atom Site Occupancy of O6         Constrained at        0.5 Check
PLAT300_ALERT_4_G Atom Site Occupancy of O7         Constrained at        0.5 Check
PLAT300_ALERT_4_G Atom Site Occupancy of O8         Constrained at        0.5 Check
PLAT300_ALERT_4_G Atom Site Occupancy of O9         Constrained at        0.5 Check
PLAT300_ALERT_4_G Atom Site Occupancy of O11        Constrained at        0.5 Check
PLAT300_ALERT_4_G Atom Site Occupancy of O15'       Constrained at        0.6 Check
PLAT300_ALERT_4_G Atom Site Occupancy of O18        Constrained at        0.5 Check
PLAT300_ALERT_4_G Atom Site Occupancy of O19        Constrained at        0.5 Check
PLAT300_ALERT_4_G Atom Site Occupancy of O15        Constrained at        0.4 Check
PLAT300_ALERT_4_G Atom Site Occupancy of C2         Constrained at        0.5 Check
PLAT300_ALERT_4_G Atom Site Occupancy of C3         Constrained at        0.5 Check
PLAT300_ALERT_4_G Atom Site Occupancy of C4         Constrained at       0.25 Check
PLAT300_ALERT_4_G Atom Site Occupancy of Se3'       Constrained at       0.25 Check
PLAT300_ALERT_4_G Atom Site Occupancy of O24        Constrained at        0.2 Check
PLAT300_ALERT_4_G Atom Site Occupancy of O24'       Constrained at       0.26 Check
PLAT300_ALERT_4_G Atom Site Occupancy of O21        Constrained at       0.48 Check
PLAT300_ALERT_4_G Atom Site Occupancy of O22        Constrained at       0.26 Check
PLAT300_ALERT_4_G Atom Site Occupancy of O22'       Constrained at        0.4 Check
PLAT300_ALERT_4_G Atom Site Occupancy of O22"       Constrained at       0.28 Check
PLAT300_ALERT_4_G Atom Site Occupancy of O23        Constrained at        0.4 Check
PLAT300_ALERT_4_G Atom Site Occupancy of O23'       Constrained at        0.2 Check
PLAT301_ALERT_3_G Main Residue  Disorder .............(Resd  1  )         73% Note
PLAT302_ALERT_4_G Anion/Solvent/Minor-Residue Disorder (Resd  2  )       100% Note
PLAT302_ALERT_4_G Anion/Solvent/Minor-Residue Disorder (Resd  3  )       100% Note
PLAT302_ALERT_4_G Anion/Solvent/Minor-Residue Disorder (Resd  4  )       100% Note
PLAT302_ALERT_4_G Anion/Solvent/Minor-Residue Disorder (Resd  5  )       100% Note
PLAT302_ALERT_4_G Anion/Solvent/Minor-Residue Disorder (Resd  6  )       100% Note
```

```
PLAT302_ALERT_4_G Anion/Solvent/Minor-Residue Disorder (Resd  7  )       100% Note
PLAT302_ALERT_4_G Anion/Solvent/Minor-Residue Disorder (Resd  8  )       100% Note
PLAT302_ALERT_4_G Anion/Solvent/Minor-Residue Disorder (Resd  9  )       100% Note
PLAT304_ALERT_4_G Non-Integer Number of Atoms in ..... (Resd  1  )     139.38 Check
PLAT304_ALERT_4_G Non-Integer Number of Atoms in ..... (Resd  2  )       0.50 Check
PLAT304_ALERT_4_G Non-Integer Number of Atoms in ..... (Resd  3  )       0.23 Check
PLAT304_ALERT_4_G Non-Integer Number of Atoms in ..... (Resd  4  )       0.12 Check
PLAT304_ALERT_4_G Non-Integer Number of Atoms in ..... (Resd  5  )       0.13 Check
PLAT304_ALERT_4_G Non-Integer Number of Atoms in ..... (Resd  6  )       0.10 Check
PLAT304_ALERT_4_G Non-Integer Number of Atoms in ..... (Resd  7  )       0.14 Check
PLAT304_ALERT_4_G Non-Integer Number of Atoms in ..... (Resd  8  )       0.10 Check
PLAT304_ALERT_4_G Non-Integer Number of Atoms in ..... (Resd  9  )       0.05 Check
PLAT311_ALERT_2_G Isolated Disordered Oxygen Atom (No H's ?) .....         O4 Check
PLAT311_ALERT_2_G Isolated Disordered Oxygen Atom (No H's ?) .....         O5 Check
PLAT311_ALERT_2_G Isolated Disordered Oxygen Atom (No H's ?) .....        O24 Check
PLAT311_ALERT_2_G Isolated Disordered Oxygen Atom (No H's ?) .....       O24' Check
PLAT311_ALERT_2_G Isolated Disordered Oxygen Atom (No H's ?) .....        O21 Check
PLAT311_ALERT_2_G Isolated Disordered Oxygen Atom (No H's ?) .....        O22 Check
PLAT311_ALERT_2_G Isolated Disordered Oxygen Atom (No H's ?) .....       O22' Check
PLAT311_ALERT_2_G Isolated Disordered Oxygen Atom (No H's ?) .....       O22" Check
PLAT311_ALERT_2_G Isolated Disordered Oxygen Atom (No H's ?) .....        O23 Check
PLAT311_ALERT_2_G Isolated Disordered Oxygen Atom (No H's ?) .....       O23' Check
PLAT606_ALERT_4_G Solvent Accessible VOID(S) in Structure ........          ! Info
PLAT773_ALERT_2_G Check long C-C Bond in CIF: C4       --C4            1.80 Ang.
PLAT811_ALERT_5_G No ADDSYM Analysis: Too Many Excluded Atoms ....          ! Info
PLAT860_ALERT_3_G Number of Least-Squares Restraints ............         154 Note
PLAT869_ALERT_4_G ALERTS Related to the Use of SQUEEZE  Suppressed          ! Info
PLAT913_ALERT_3_G Missing # of Very Strong Reflections in FCF ....          1 Note
PLAT952_ALERT_5_G Calculated (ThMax) and CIF-Reported Lmax Differ.          2 Units
PLAT955_ALERT_1_G Reported (CIF) and Actual (FCF) Lmax Differ by .          2 Units
PLAT965_ALERT_2_G The SHELXL WEIGHT Optimisation has not Converged     Please Check
```

---

0 **ALERT level A** = Most likely a serious problem - resolve or explain
3 **ALERT level B** = A potentially serious problem, consider carefully
15 **ALERT level C** = Check. Ensure it is not caused by an omission or oversight
91 **ALERT level G** = General information/check it is not something unexpected

6 ALERT type 1 CIF construction/syntax error, inconsistent or missing data
28 ALERT type 2 Indicator that the structure model may be wrong or deficient
16 ALERT type 3 Indicator that the structure quality may be low
57 ALERT type 4 Improvement, methodology, query or suggestion
2 ALERT type 5 Informative message, check

It is advisable to attempt to resolve as many as possible of the alerts in all categories. Often the minor alerts point to easily fixed oversights, errors and omissions in your CIF or refinement strategy, so attention to these fine details can be worthwhile. In order to resolve some of the more serious problems it may be necessary to carry out additional measurements or structure refinements. However, the purpose of your study may justify the reported deviations and the more serious of these should normally be commented upon in the discussion or experimental section of a paper or in the "special_details" fields of the CIF. checkCIF was carefully designed to identify outliers and unusual parameters, but every test has its limitations and alerts that are not important in a particular case may appear. Conversely, the absence of alerts does not guarantee there are no aspects of the results needing attention. It is up to the individual to critically assess their own results and, if necessary, seek expert advice.

Publication of your CIF in IUCr journals

A basic structural check has been run on your CIF. These basic checks will be run on all CIFs submitted for publication in IUCr journals (*Acta Crystallographica*, *Journal of Applied Crystallography*, *Journal of Synchrotron Radiation*); however, if you intend to submit to *Acta Crystallographica Section C* or *E* or *IUCrData*, you should make sure that full publication checks are run on the final version of your CIF prior to submission.

Publication of your CIF in other journals

Please refer to the *Notes for Authors* of the relevant journal for any special instructions relating to CIF submission.

PLATON version of 28/11/2022; check.def file version of 28/11/2022

Datablock ae448i_sq - ellipsoid plot

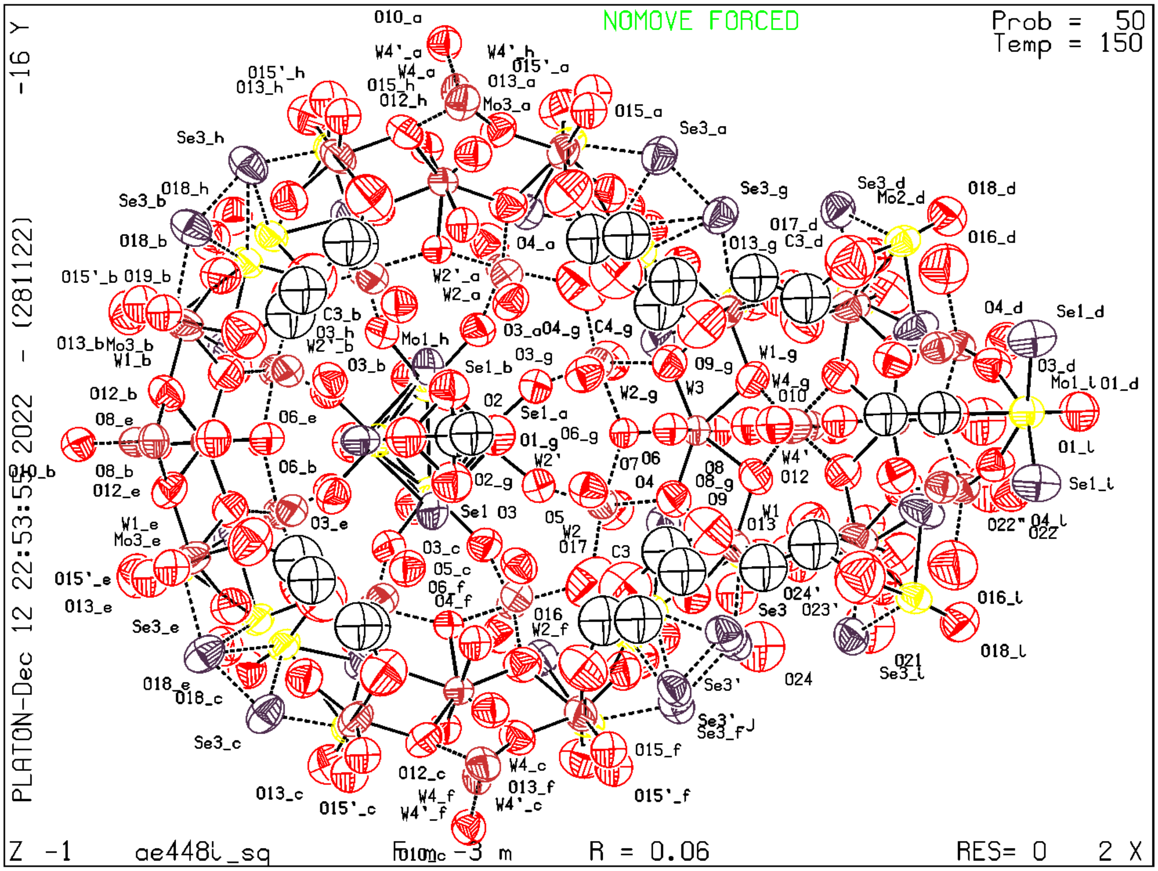

Supplement: Supplementary file 4 — Supporting Information [file ANIE-62-0-s004.pdf]
