# Supplementary material for: Pore “Softening” and Emergence of Breathability Effects of New Keplerate Nano‐Containers
Source: Angew Chem Int Ed Engl. 2023 Mar 9;62(20):e202218897. doi: 10.1002/anie.202218897 (PMC10946700; doi:10.1002/anie.202218897)
Supplement: Supplementary file 5 — Supporting Information [file ANIE-62-0-s003.pdf]

Angewandte Chemie
Eine Zeitschrift der Gesellschaft Deutscher Chemiker

Supporting Information

Pore "Softening" and Emergence of Breathability Effects of New Keplerate Nano-Containers

A. Elliott, J. McAllister, D.-L. Long, Y.-F. Song*, H. N. Miras**

Table of contents

1.	Synthetic procedures	S3
2.	Pore size and molecule diameter measurements	S5
3.	Ligands used	S7
4.	UV/Vis measurement	S8
5.	Elemental Analysis	S9
6.	NMR studies	S10
6.1.	Ligand exchange experiments	S10
6.2.	DOSY	S11
6.3.	Ligand separation experiments	S12
7.	Infrared spectroscopy (FT-IR)	S20
8.	Crystal structure data	S22
9.	PXRD	S23
10.	Thermal gravimetric analysis (TGA)	S24
11.	Kinetic Model	S28
12.	References	S30

1. Synthetic procedures

Synthesis of $[Mo_{132}Se_{60}O_{312}(H_2O)_{72}(AcO)_{30}]^{42-}$, $[W_{72}Mo_{60}Se_{60}O_{312}(H_2O)_{72}(AcO)_{30}]^{42-}$, and $[Mo_{132}S_{60}O_{312}(H_2O)_{72}(AcO)_{30}]^{42-}$

Selenide Keplerates are synthesised based on a modified method reported by Bannani *et al*[1] for the synthesis of sulphide Keplerates.

$[Mo_{132}Se_{60}O_{312}(H_2O)_{72}(AcO)_{30}]^{42-}$ (1): $Na_2MoO_4.2H_2O$ (150 mg, 0.620 mmol) and anhydrous sodium acetate (1.8 g, 21.9 mmol) are dissolved in a solution of H_2O (6 mL) and glacial acetic acid (4 mL). $[(Mo_2O_2Se_2(OH)_2(H_2O))_5I_2]K_x(NMe_4)_{2-x}$ (220 mg) is hydrolysed in 3 mL of 4 M HCl for 15 min at 50°C then filtered into the molybdate solution. The pH was adjusted to 3.55 via addition of glacial acetic acid. The solution was allowed to stand for precipitation. The precipitate was collected under reduced pressure and washed with ethanol and diethyl ether. The sample was purified for analysis by redissolving in a minimum of water and reprecipitation with tetramethyl ammonium chloride. Elemental analysis calculated for $Na_{12}(NMe_4)_{30}[Mo_{132}Se_{60}O_{312}(H_2O)_{72}(AcO)_{30}](H_2O)_{80}$, Mw = 29403 g mol^{-1}: C 7.35, H 2.58, N 1.43, Mo 43.10, Se 16.11; found C 7.22, H 2.40, N 1.64, Mo 43.19, Se 18.16. Large single crystals suitable for X-ray diffraction were obtained by further recrystallisation in an ammonium chloride solution. Overall yield 33.7 mg (13.3% based on Mo). Elemental analysis calculated for $(NH_4)_{42}[Mo_{132}Se_{60}O_{312}(H_2O)_{72}(AcO)_{30}](H_2O)_{200}$, Mw = 29822 g mol^{-1}: C 2.42, H 2.71, N 1.97, Mo 42.46, Se 15.88; found: C 1.75, H 2.07, N 2.25, Mo 43.36, Se 14.80.

$[W_{72}Mo_{60}Se_{60}O_{312}(H_2O)_{72}(AcO)_{30}]^{42-}$ (2): The same method is used as for **1**, except $Na_2WO_4.2H_2O$ (200 mg, 0.606 mmol) is used in place of $Na_2MoO_4.2H_2O$. Final product was obtained by recrystallisation in an ammonium chloride solution. Overall yield 123 mg (19.8% based on W). Elemental analysis calculated for $(NH_4)_{42}[W_{72}Mo_{60}Se_{60}O_{312}(H_2O)_{72}(AcO)_{30}](H_2O)_{200}$, Mw = 36152 g/mol: C 1.99, H 2.23, N 1.63, Mo 15.92, W 36.61, Se 13.10; found: C 2.21, H 2.15, N 2.14; Mo 16.87, W 36.95, Se 12.22.

$[Mo_{132}S_{60}O_{312}(H_2O)_{72}(AcO)_{30}]^{42-}$ (3): Synthesised following previously reported method by Cadot *et al*.[1]

$[Mo_{132}O_{372}(H_2O)_{72}(AcO)_{30}]^{42-}$ (4): Oxygenated Keplerate is synthesised *via* a typical method of reducing a solution of molybdate in acetate buffer.[2] In full $Na_2MoO_4.2H_2O$ (3g, 12 mmol) and $Na_2S_2O_4$ (0.45 g, 2.58 mmol) were dissolved in 80 mL of an ammonium acetate buffer consisting of a 3:1 ratio of water to glacial acetic acid to which 25 wt% ammonium hydroxide was added to reach pH 4. The solution thus prepared was stirred until it turned red/brown, and then filtered and allowed to stand for two days. The solid obtained was collected *via* vacuum filtration and washed with ethanol and diethyl ether. Crystal identity was verified by unit cell comparison.

$[Mo_2(\mu\text{-}Se)_2O_2(Se_2)_2](NMe_4)_2$ (5): Selenide dimer was synthesised *via* our previously reported synthesis.[3] In a typical synthesis selenium (4.738 g, ~60 mmol) was suspended in 5% ammonia solution (200 mL) at heated to 40°C. Nitrogen is bubbled into this solution and the exhaust gas is passed through a bleach trap. Sodium borohydride (473 mg, 12.5 mmol) is dissolved in water (25 mL) whilst cooling in an ice bath. The solution is degassed by bubbling nitrogen for at least 10 minutes. The borohydride solution is slowly added to the suspension of selenium turning a dark red colour. Once added the temperature is increased to 50°C and the solution stirred for a further 30 minutes to ensure the borohydride reacts fully. A solution of ammonium molybdate tetrahydrate (3.531 mg, 20 mmol Mo) is dissolved in water (50 mL) and degassed by bubbling nitrogen through for at least 10 minutes. This solution is then

transferred to the selenium solution. Tetramethylammonium chloride (5 g, 45.6 mmol) is then dissolved in water (5 mL) and degassed in the same way, which is then added following the ammonium molybdate tetrahydrate. The solution is allowed to cool to room temperature and is stirred overnight. A dark brown/red precipitate is then collected via vacuum filtration and washed with water, cold ethanol and diethyl ether.

$[(Mo_2O_2Se_2(OH)_2(H_2O))_5I_2]K_x(NMe_4)_{2-x}$: In a typical synthesis solid **5** (1 g, ~1.18 mmol) is suspended in water (10 mL). Iodine (0.9 g, 3.55 mmol) is dissolved in 1M hydrochloric acid (15 mL), and ammonium iodide (1.25 g, 8.62 mmol) is added to aid dissolution. Both solutions are degassed with nitrogen. The iodine solution is then added slowly to **5** with stirring and following complete addition the solution is heated to $50^{O}C$ and stirred for 15 minutes. The solution thus obtained is then filtered whilst hot, cooled in a fridge for at least 1 hour, then filtered a second time. Potassium hydroxide solution (2M) is added to the filtered solution until it reaches a pH of 2. The solution is then stirred for 1 hour and allowed to precipitate in a fridge overnight. The precipitate is collected *via* vacuum filtration and washed with ice cold water, cold ethanol, and diethyl ether.

2. Pore size and molecule diameter measurements

The measurements of pore radius and area, alongside the diameter of **1**, **3** and **4** which are summarised in Table 1 are shown here graphically in figure S1 a). The pore radius is determined from the E-E chalcogenide spacing within the pore, along with standard values of the Van der Waals radius of the chalcogenide using the equation $r_{pore} = \frac{\sqrt{3}}{3} l - r_{vdw}$ where r_{pore} is the pore radius, l is the E-E spacing in the pore and r_{vdw} is the Van der Waals radius. The diameter is measured as the spacing between the central Mo^{VI} atoms of opposite pentagonal units. In each case an average of all such measurements within the structure is used.

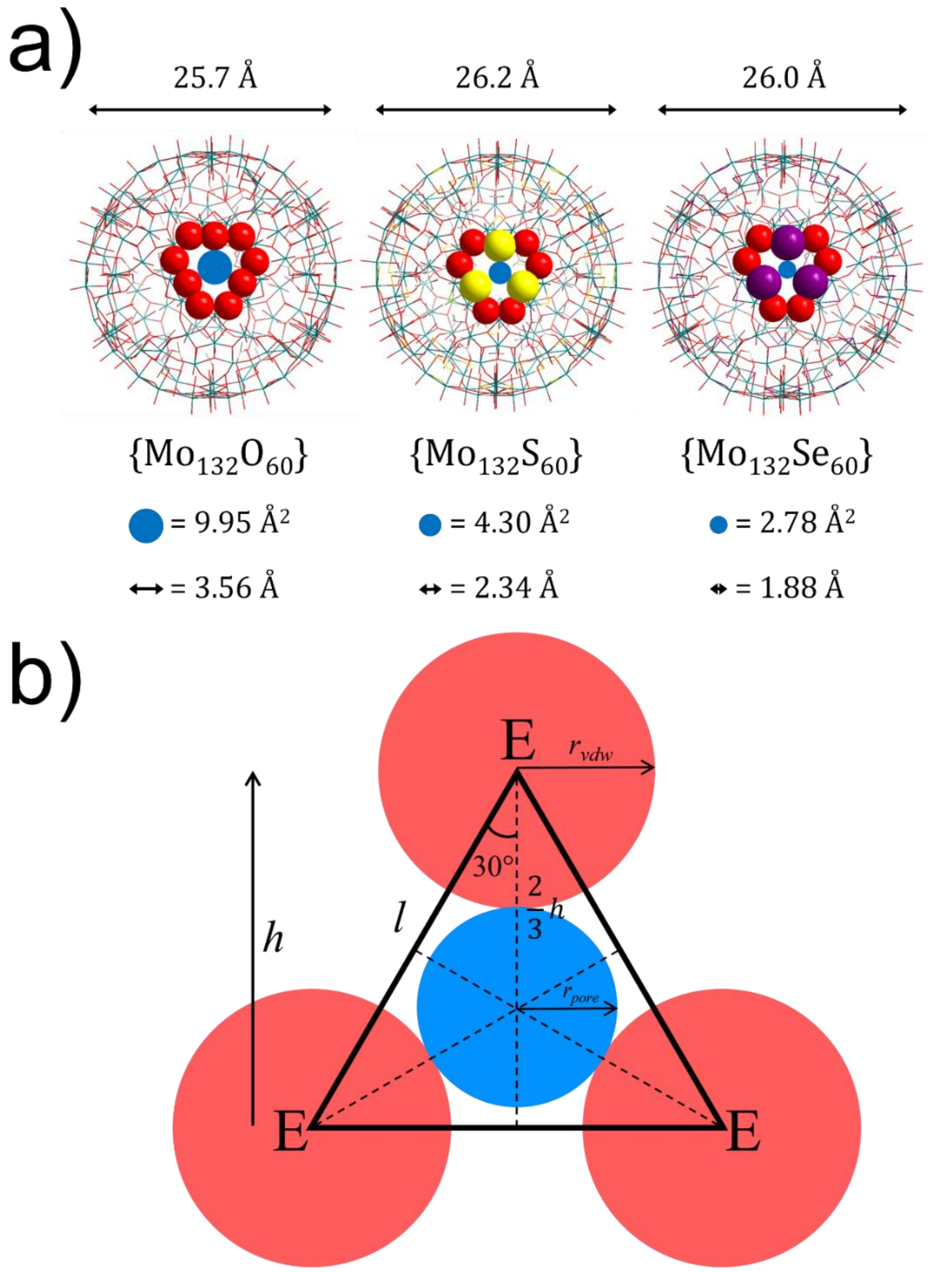

c)

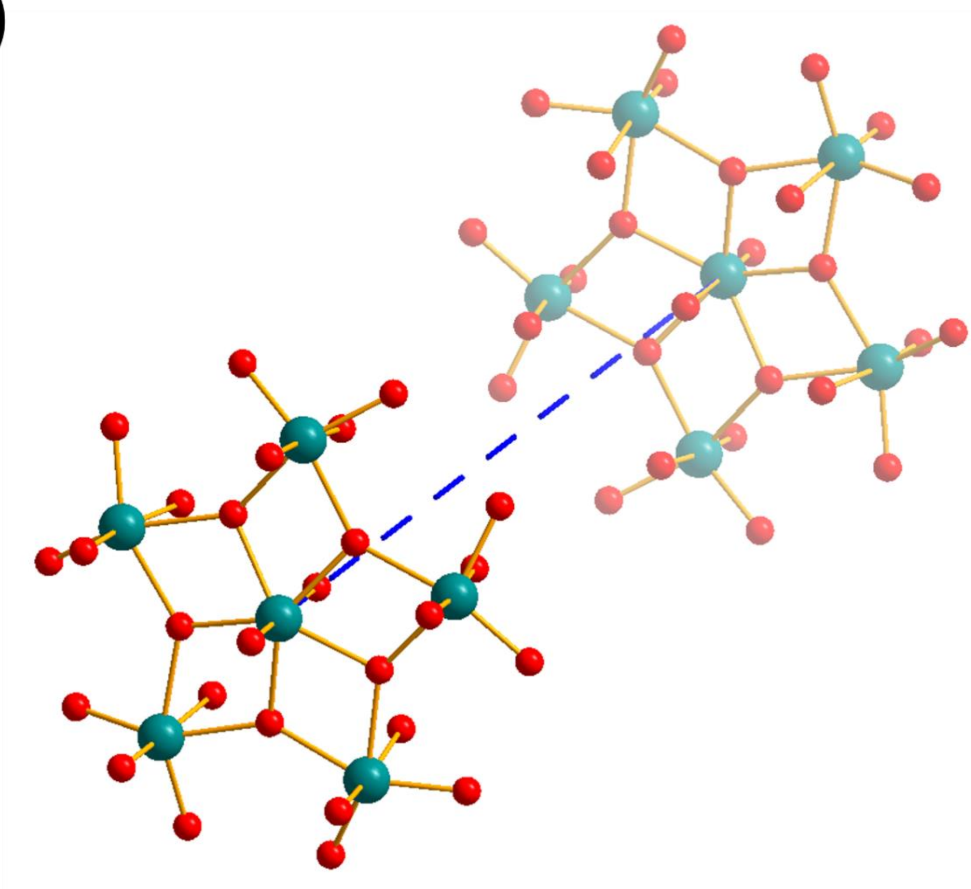

Figure S1: a) graphical representation of the diameter, pore diameter and pore area of **1**, **3** and **4**, b) schematic representation of how the circular pore area is determined based on three chalcogenide spheres E arranged in an equilateral triangle defining the pore area and c) illustration of the pairs of Mo atoms (indicated by dashed blue line) which are used to determine the diameter of the Keplerate.

3. Ligands used

Structures of the three organic ligands used are shown here, along with their informal names.

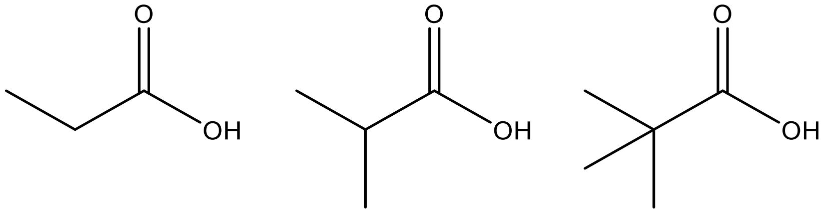

Propionic Acid **Isobutyric Acid** **Pivalic Acid**

4. UV/Vis measurement

10 mg of **1** was dissolved in 25 mL of 0.2 M isobutyric acid (^{i}PrCOOH) solution. This solution was kept at room temperature over several weeks in order to monitor stability of **1** in conditions analogous to the ligand exchange experiments. UV/Vis measurements were made by removing 2 mL and diluting in a 1:4 ratio before measuring the spectrum between 300 nm and 900 nm on a Jasco V-670 Spectrophotometer at a scan speed of 400 nm min^{-1} and a bandwidth of 0.5 nm in the UV/Vis range and 2 nm in the NIR range.

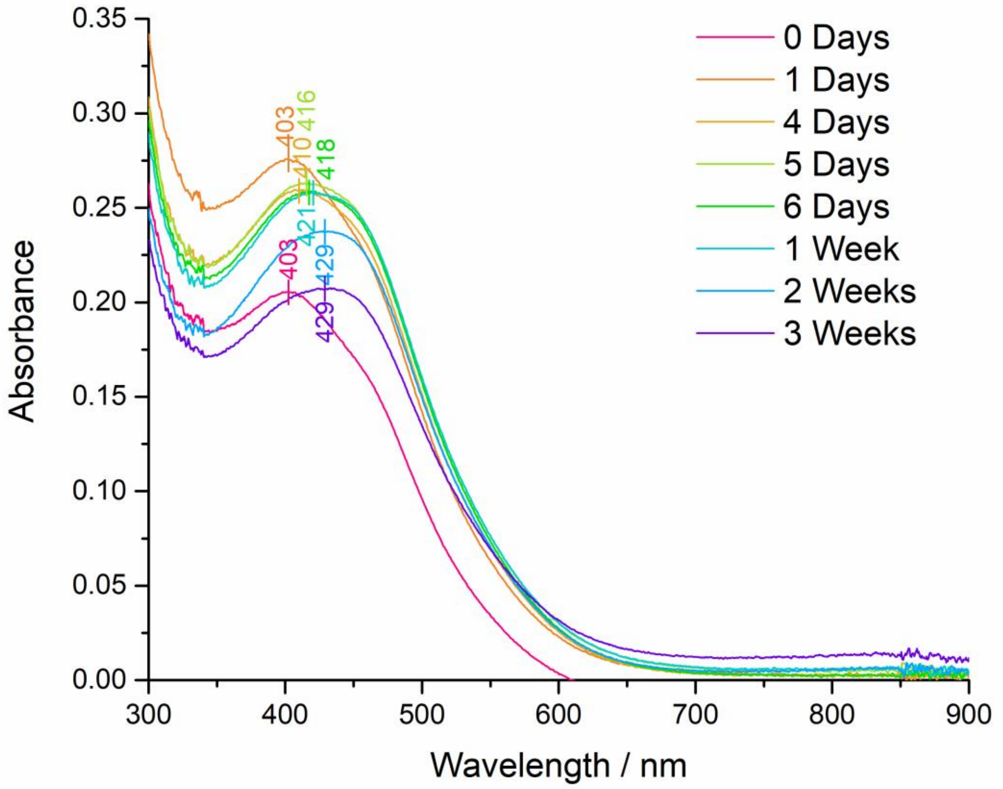

Figure S2: UV/Vis measurement of **1** in a solution of carboxylic acid analogous to ligand exchange solutions. Over the course of 2 weeks the peak position shifts from 403 nm to 429 nm, however it is constant at 403 nm for the first day. After reaching 429 nm the peak does not shift after another week.

It is evident the stability of the Keplerate structure for the duration of the investigation. Rapid breakdown of the capsule would decrease the available number of sites, and hence the number of attached ligands, leading to a decrease in the equilibrium number of encapsulated ligands, giving the appearance that a near equilibrium state is reached more rapidly, which would appear to give similar results to those obtained. The stability of **1** was explored in exchange conditions (i.e. in the presence of unbuffered organic acid) via UV/vis spectroscopy. Over the course of 24 hours the peak maximum did not shift in first day indicating that a chemical transformation has not yet occurred at this point. In subsequent days the peak position shifted by 26 nm over the course of 2 weeks, likely indicative of a change in the chemical environment around the Mo^{V} which is responsible for this peak suggesting a minor degree of decomposition within this timeframe.

5. Elemental analysis

Analysis of Mo and Se performed via ICP-OES using an Agilent 5100 ICP-OES calibrated using commercially purchased molybdenum and selenium standards. Samples of approximately 5 mg were digested in a 2:1 mixture of nitric and sulphuric acid with gentle heating in a sealed container. Once digested the container was vigorously shaken in order to oxidise any H_2Se that may have formed during digestion. Afterwards ICP grade H_2O is carefully added to dilute to 50 mL.

6. NMR studies

All NMR studies were performed on a Bruker Avance III HD 600 spectrometer. 1D ^{1}H spectra used standard pulse sequences. DOSY measurements used 16 slices and a total of 128 scans. The gradient pulse was set to 1500 µsec, all other parameters were unmodified from the typical DOSY pulse sequence.

6.1. Single ligand exchange experiments

In a typical 5 mm NMR tube is layered a solution of solid Keplerate salt in D_2O (4 mg of solid dissolved in 0.2 mL D_2O) followed by 0.2 mL D_2O as a spacer and then 0.2 mL of a 0.2 M solution of ligand in D_2O. The NMR tube is mixed within one minute of the first measurement by inverting several times until a uniform colour is achieved. To confirm the results each experiment was repeated twice and show the same trend.

To account for differences in concentration of Keplerate and number of available ligand exchange sites the fractional reaction progress is calculated. This is determined by the ratio of the current fraction of ligands encapsulated to the total fraction of ligands encapsulated at the end of the timescale studied. Faster ligand exchange will therefore lead to more rapid initial reaction progress and a faster approach to equilibrium; therefore, the reaction progress will increase faster initially, and level off earlier than for slow ligand exchange. In this way relative exchange rates can be compared qualitatively.

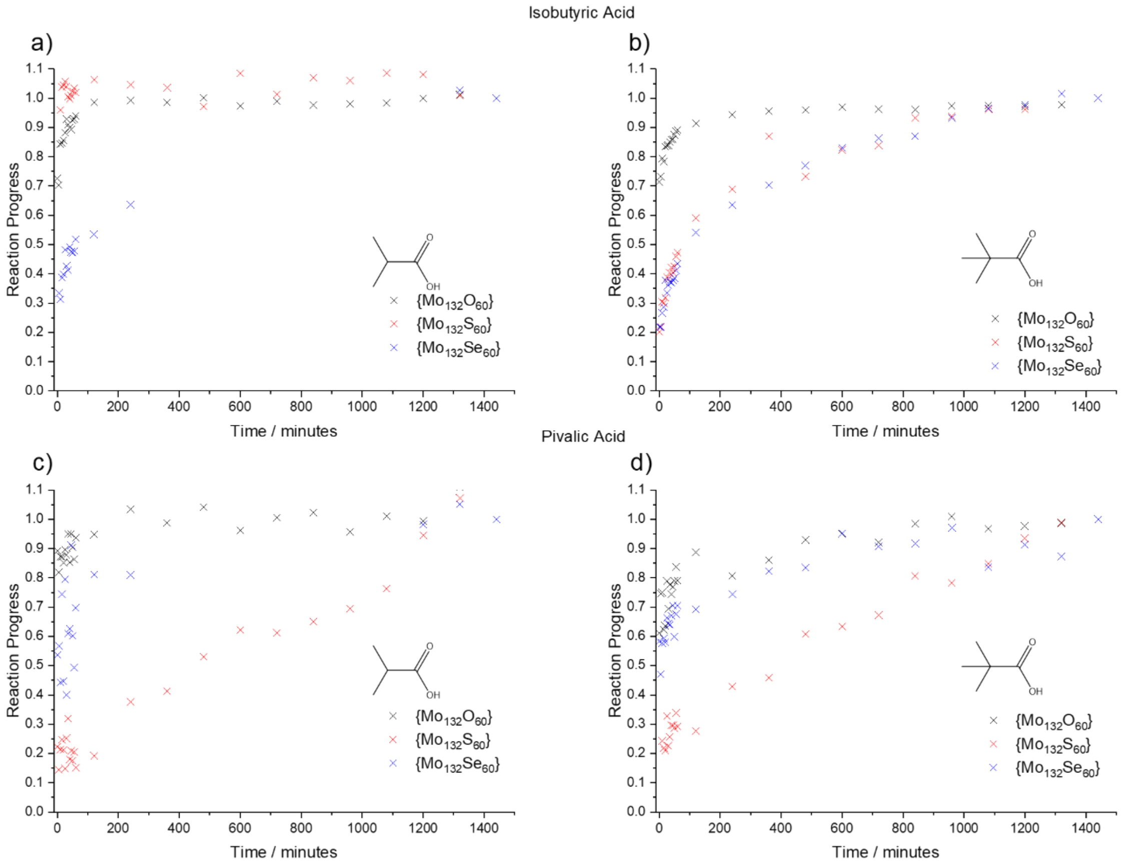

Figure S3: Repeats of ligand exchange experiments over the course of 1 day. a) and b) isobutyric acid, c) and d) pivalic acid.

Interpretation of NMR data

The broad peak located between -0.35 ppm and 0.16 ppm is attributed to encapsulated methyl protons. These appear distinct from all other signals in the spectrum and thus make for easy quantification. As the total number of ligand molecules present in solution is fixed it is assumed that the sum of the integral of the encapsulated methyl protons I_{inside} and the integral of the free methyl protons $I_{outside}$ is constant. The fraction of ligands encapsulated can therefore be estimated as $\frac{I_{inside}}{I_{inside}+I_{outside}}$, by normalising $I_{inside} = 1$ this reduces to $\frac{1}{1+I_{outside}}$.

6.2. DOSY

An NMR tube is prepared containing 4 mg of **4** and 4 mg of pivalic acid in 0.6 mL D_2O and allowed to exchange for over a week. The resulting solution is examined using DOSY. Free acetic acid (at ~2 ppm) diffuses at approximately $2x10^{-5}$ cm^2s^{-1} and pivalic acid (at ~1.1 ppm) diffuses at approximate $4x10^{-5}$ cm^2s^{-1}, whereas the peaks attributed to encapsulated acetate (0.6 ppm) and encapsulated (-0.2 ppm) diffuse at approximately $4x10^{-6}$ cm^2s^{-1} and $2x10^{-6}$ cm^2s^{-1} respectively, providing unambiguous evidence that these peaks are located within a large (slowly diffusing) capsule such as a Keplerate. The relatively faster diffusion of pivalic acid is due to the greater degrees of freedom available to the protons which are located on C-C bond further away from the anchor point.

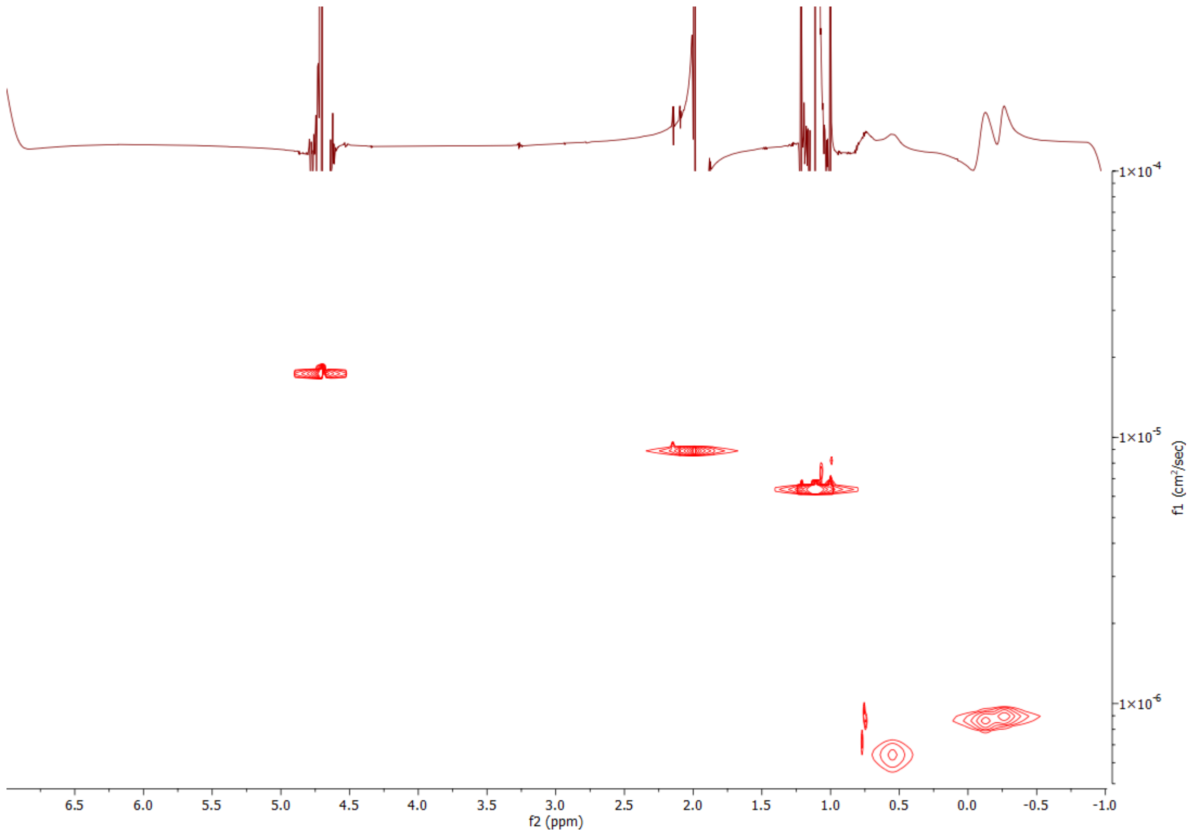

Figure S4: DOSY of **4** in the presence of pivalic acid. The x axis shows chemical shift in ppm whilst the y axis shows approximate diffusion speed in cm^2 s^{-1}

6.3. Ligand separation experiments

Preparation of samples

Prior to these experiments all the Keplerate cages were stirred in water overnight to remove any excess acetate that may be crystallised alongside the Keplerate, and to leach as much acetate from within the cage as possible. They were reprecipitated by addition of excess ammonium chloride.

In the 'ligand mixture' experiments a stock solution of 20 mM propionic acid, 20 mM pivalic acid, 40 mM lithium hydroxide and 10 mM benzenetricarboxylic acid was prepared in deuterated water, the final pH being approximately 4.5. 4 mg of Keplerate was dissolved 600 µL of this stock solution and stirred for 30 minutes in a sealed container. This solution was then filtered into an NMR tube. The first measurement was taken approximately 24 hours after preparation, and subsequent measurements were taken every 24 hours for 14 days.

In the 'ligand extraction' experiments 20 mg of Keplerate was dissolved in 1 mL of non-deuterated stock solution containing 0.05 M propionic acid and 0.05 M pivalic acid and stirred for 30 minutes in a sealed container. An excess of ethanol (typically 9 mL) was added to induce rapid precipitation of the Keplerate (the two ligands being highly soluble/miscible in ethanol would not be expected to precipitate) which was isolated by centrifuge and washed once with ethanol and twice with ether. 4 mg of the solid material thus obtained was then redissolved in 600 µL of deuterated water, and a 50 µL solution containing 5 mg LiOH in order to disintegrate the capsule and 5 mg Fumaric acid as a calibrant was added, stirring briefly to induce a colour change from dark red to pale orange/yellow. A blank was prepared consisting of 600 µL deuterated water, 20 µL of the non-deuterated acid stock solution, and 50 µL of the LiOH/Fumaric acid solution.

Analysis of NMR data

Free propionic acid shows two peaks, one triplet corresponding to the CH_3 protons at 1.1 ppm and one quartet corresponding to the CH_2 protons at 2.4 ppm. Free pivalic acid shows only one singlet peak at 1.2 ppm. These two methyl peaks show only slight overlap. Encapsulated forms of these ligands both show an overlapping broad peak at around -0.2 from the methyl groups, whilst propionic acid also shows a second broad peak around 0.95 ppm arising from the CH_2 group. This overlaps somewhat with the free methyl peak of propionic acid, especially in the case of the selenide Keplerate that shows smaller upfield shifts than other Keplerates. Proton-proton coupling could not be resolved for these encapsulated peaks. Acetate present in the initial Keplerate is present as a singlet peak at 2.1 ppm, and encapsulated at 0.55 ppm. Tetramethylammonium present in small quantities as a counter cation to the Keplerate forms a singlet peak at 3.35 ppm, and small traces of ethanol impurities may be seen forming a triplet slightly downfield of the ligand methyl peaks at 1.175 ppm. Since many peaks lie closely enough that ^{13}C satellites overlap, all integrals are calculated without satellites, however the strongly broadened internal peaks likely include satellites, and satellites of the external methyl peaks may overlap with the adjacent non-satellite peaks. All peaks were processed using standard methods. Phase adjustment and baseline correction were performed by hand.

Benzenetricarboxylic acid was added as a calibrant to the ligand mixtures in the first set of experiments. It was selected as a calibrant due to the distance of the single proton peak (8.5 ppm) from other peaks, and the size of the molecule preventing exchange with the Keplerate. However, the relative sizes of this peak compared to others showed a surprising degree of variance in the Keplerate-containing solutions, being decreased compared to other peaks. We believe that this is due to precipitation as a result of the low solubility of the acid. Solutions were filtered prior to measurement, and appeared clear after the full 14 days, and therefore

the concentration of calibrant is at least expected to be constant across all measurements, however comparison across solutions is not possible. Instead, we chose to compare Keplerate solutions to the blank solution, on the assumption that the total number of ligands in both solutions were the same. Due to the overlap of several peaks we adopted an analysis that focused on the two peaks which were the most distinct. For propanoic acid we used the peak corresponding to the external -CH_2- group which was separate from all other peaks. For pivalic acid we used the external methyl peak. The degree of overlap with the methyl group of propanoic acid was exceedingly minor, whilst at the same time the peak was roughly 3 times the area of the pivalic peak, and as such the error introduced by overlap was reasoned to be smaller than other sources of error. These peak heights were then normalised such that the total integral of all ligand peaks, internal and external, were the same as for the blank solutions. Then, comparing the external peaks in the presence of Keplerates to the external peaks of the blank we estimated the number of ligands that were missing for the Keplerate solution, and hence the number of internal ligands for both ligand types. Such a methodology is somewhat sensitive to changes in phase adjustment and baseline correction, to which we attribute the minor variations in values from day to day, however by selecting sharp, well-defined peaks these errors were minimised.

The second set of data was analysed based on the size of the free ligand peaks present. These were compared to a constant quantity of fumaric acid which was added as a calibrant. In this way relative molarities could be compared across the series, instead of ratios, allowing for a good estimate to the total number of ligands extracted, in addition to the selectivity observed.

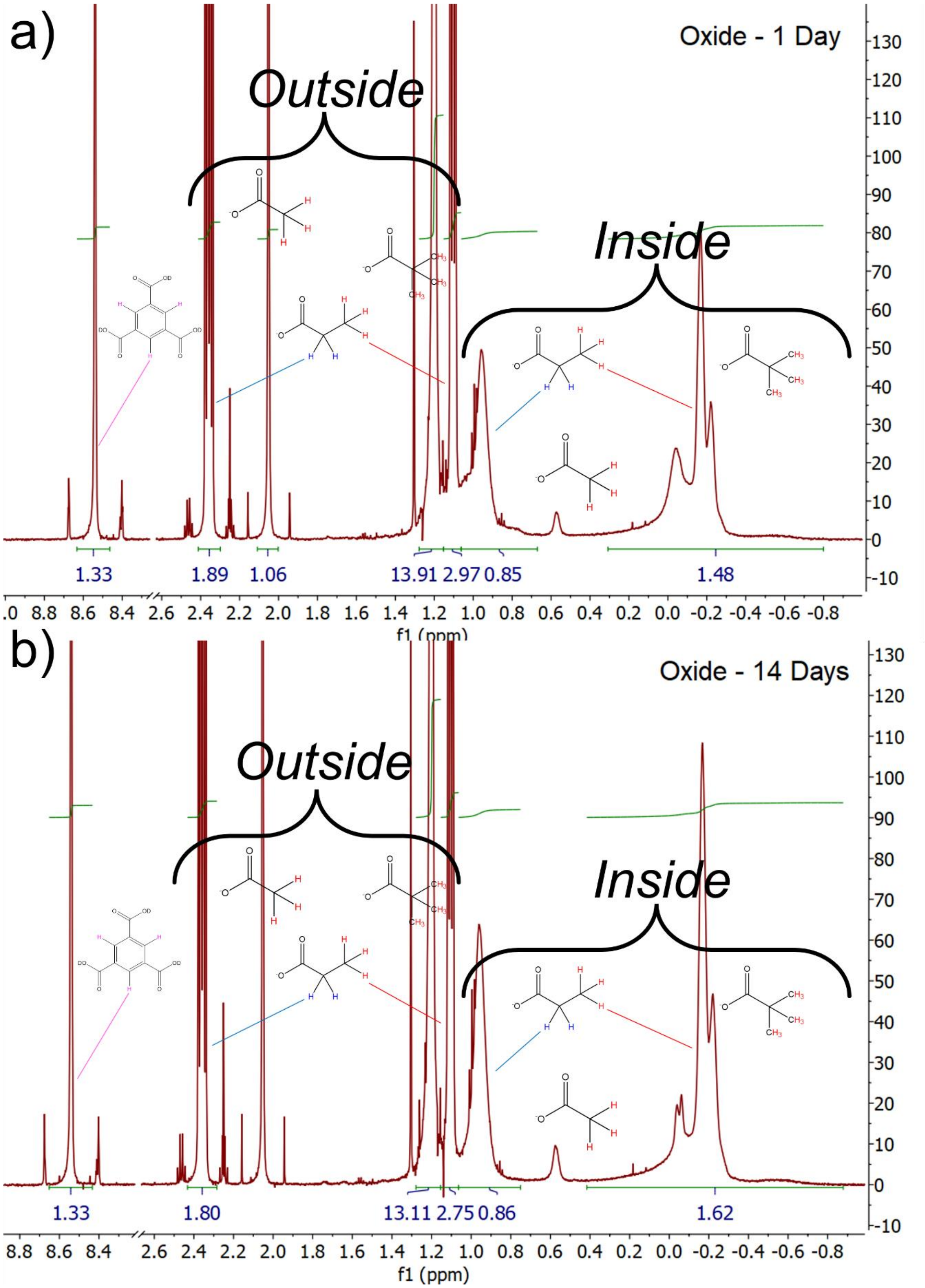

a)
Oxide - 1 Day
Outside
Inside
1.33
1.89
1.06
13.91
2.97
0.85
1.48
f1 (ppm)
b)
Oxide - 14 Days
Outside
Inside
1.33
1.80
13.11
2.75
0.86
1.62
f1 (ppm)

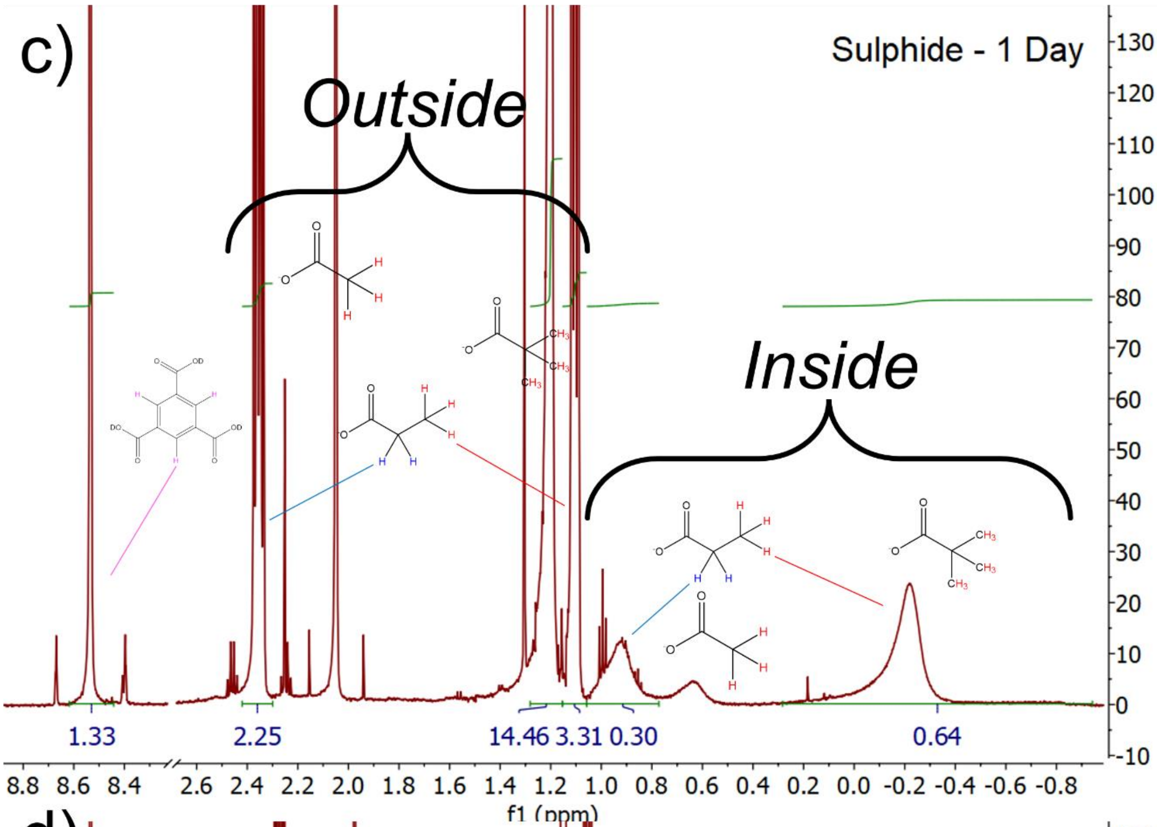
c)
Sulphide - 1 Day
Outside
Inside
1.33
2.25
14.46
3.31
0.30
0.64
f1 (ppm)

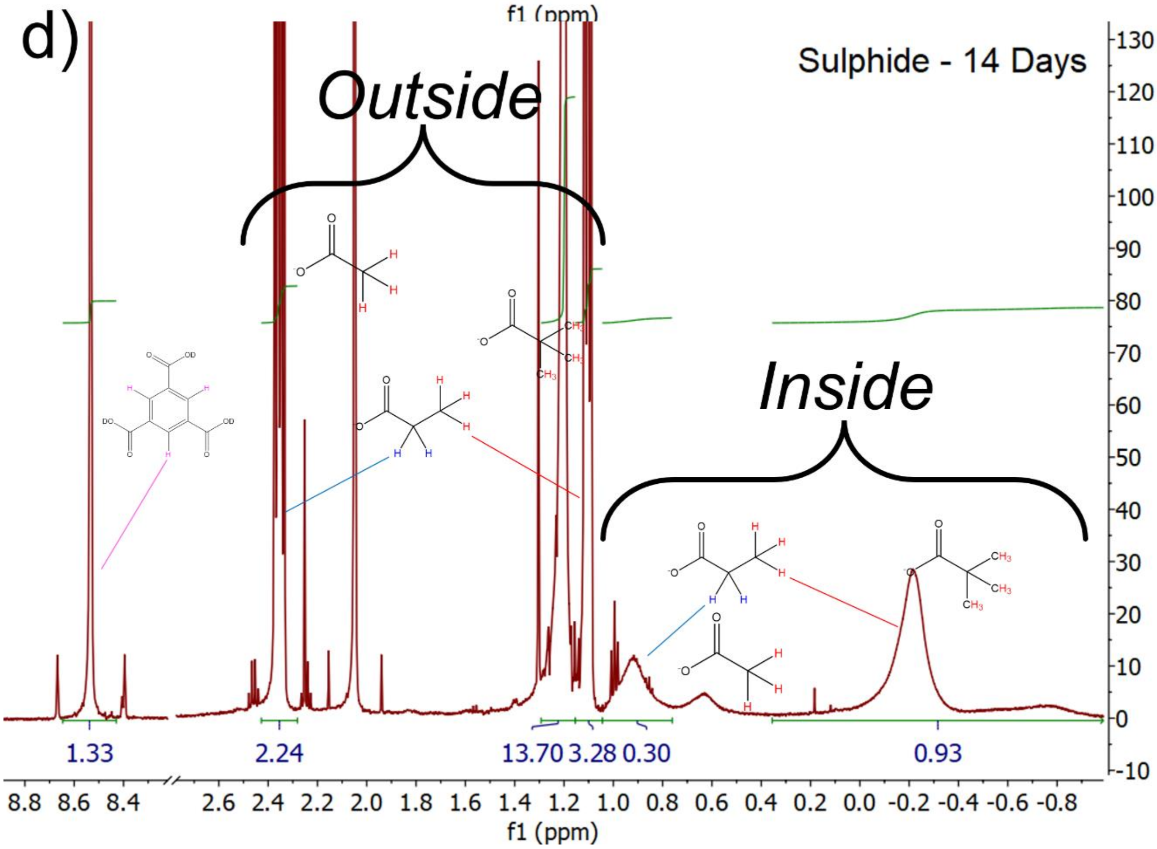
d)
Sulphide - 14 Days
Outside
Inside
1.33
2.24
13.70
3.28
0.30
0.93
f1 (ppm)

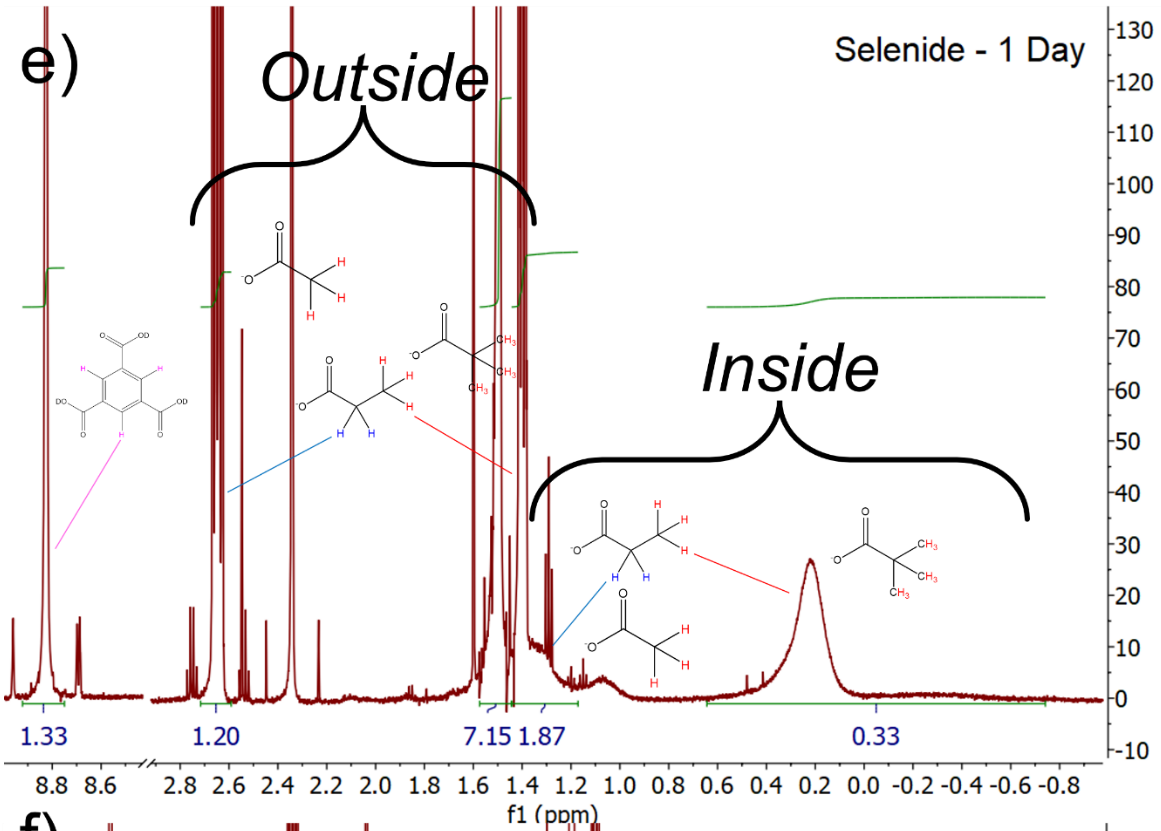

e)
Outside
Inside
Selenide - 1 Day
1.33
1.20
7.15
1.87
0.33
f1 (ppm)

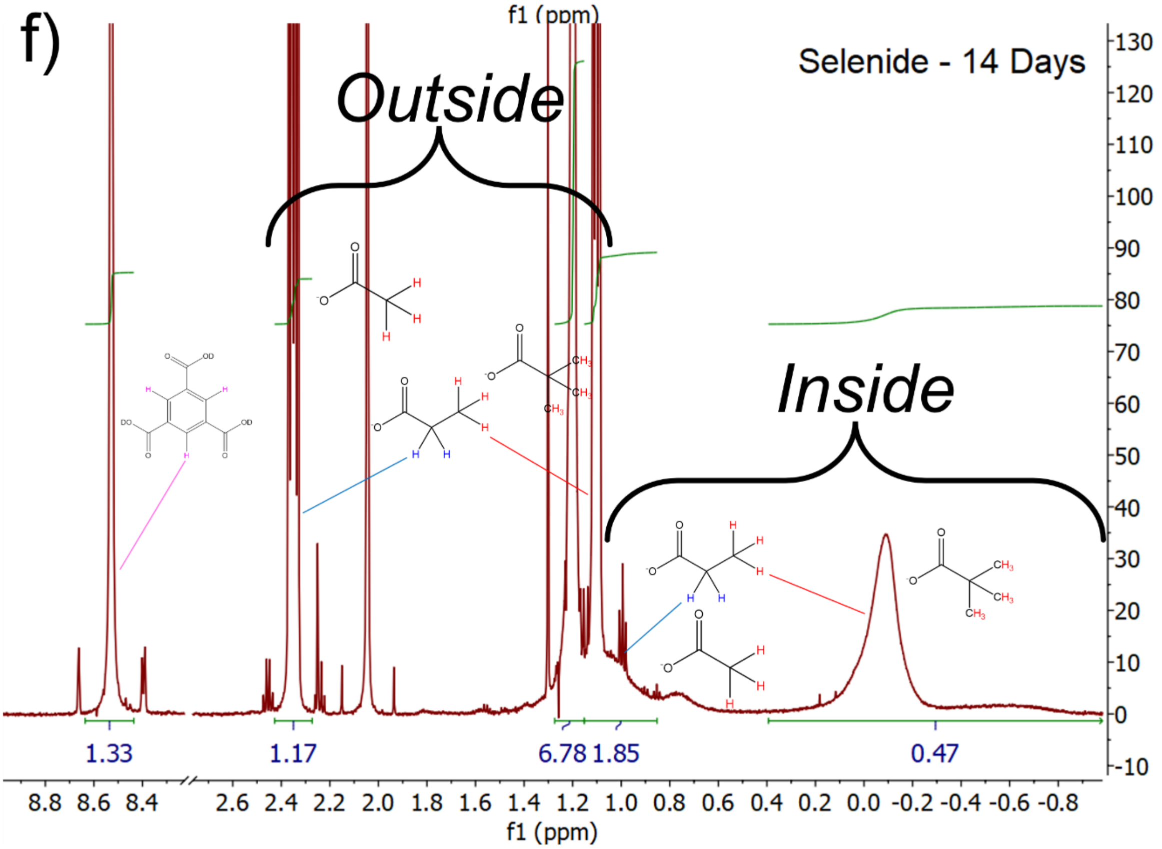

f)
Outside
Inside
Selenide - 14 Days
1.33
1.17
6.78
1.85
0.47
f1 (ppm)

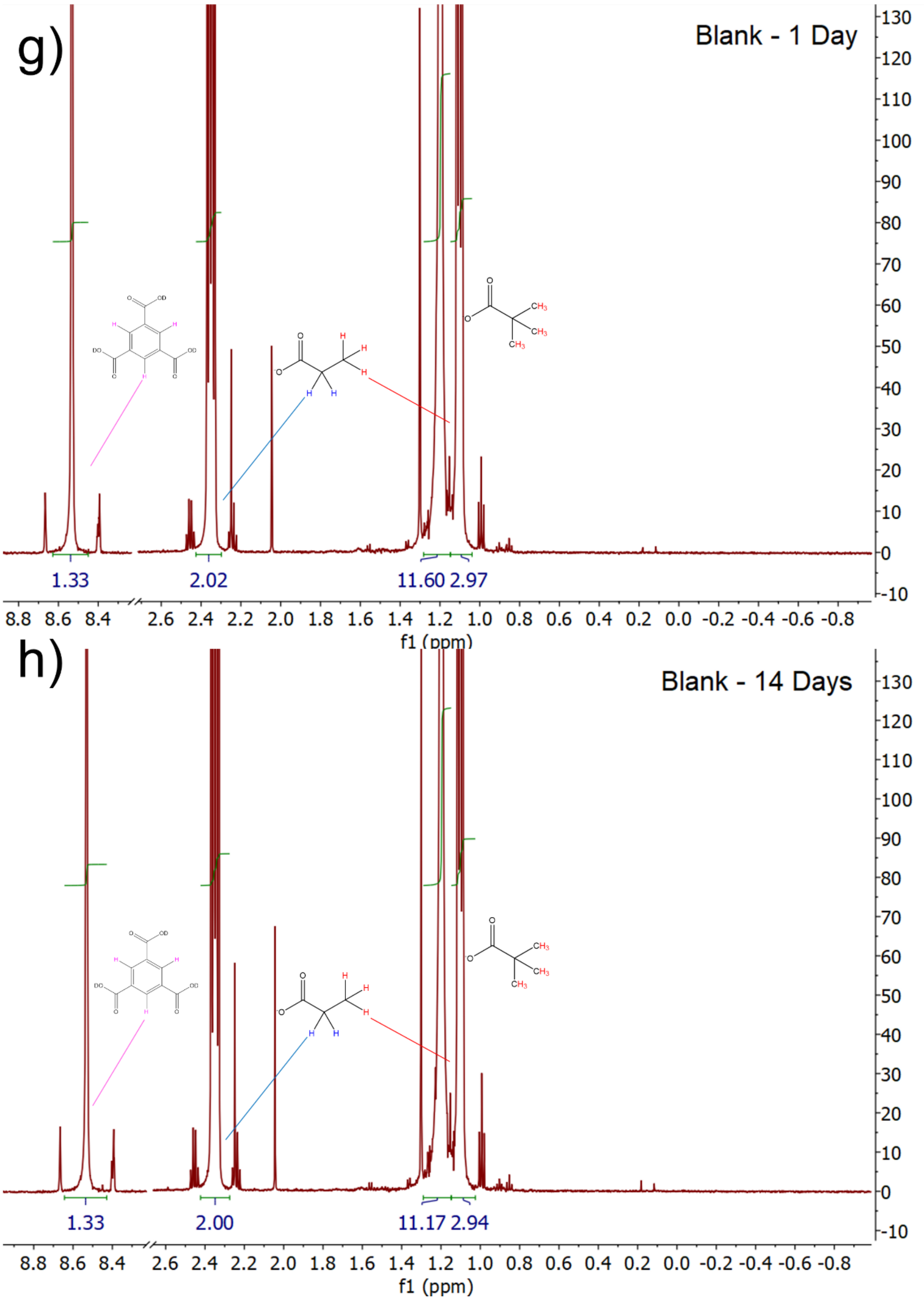

Figure S5: Representative NMR spectra of Keplerates in a mixture of ligands (a-f) after 1 and 14 days, and blank mixtures of just acids without Keplerates (g, h). Peaks are shown next to their assignment, with assigned hydrogens highlighted with colour. X axis is broken to include the calibrant at 8.5 ppm. Broad peaks below 1 ppm are assigned to ligands located within the Keplerate capsule, confirmed by previous DOSY experiments. All integrations have been normalised based on the tricarboxylic acid aromatic protons located at 8.5 ppm which is set to 1.33 protons. Integrations are calculated excluding ^{13}C satellites

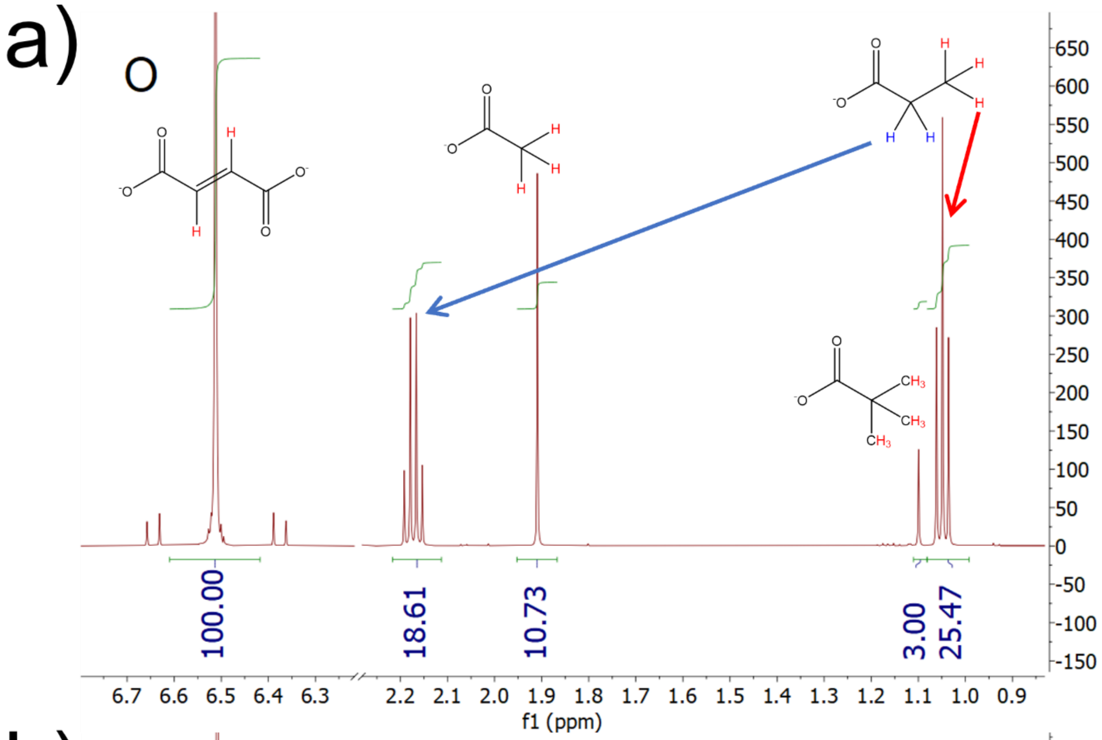
a)
O
100.00
18.61
10.73
3.00
25.47
f1 (ppm)

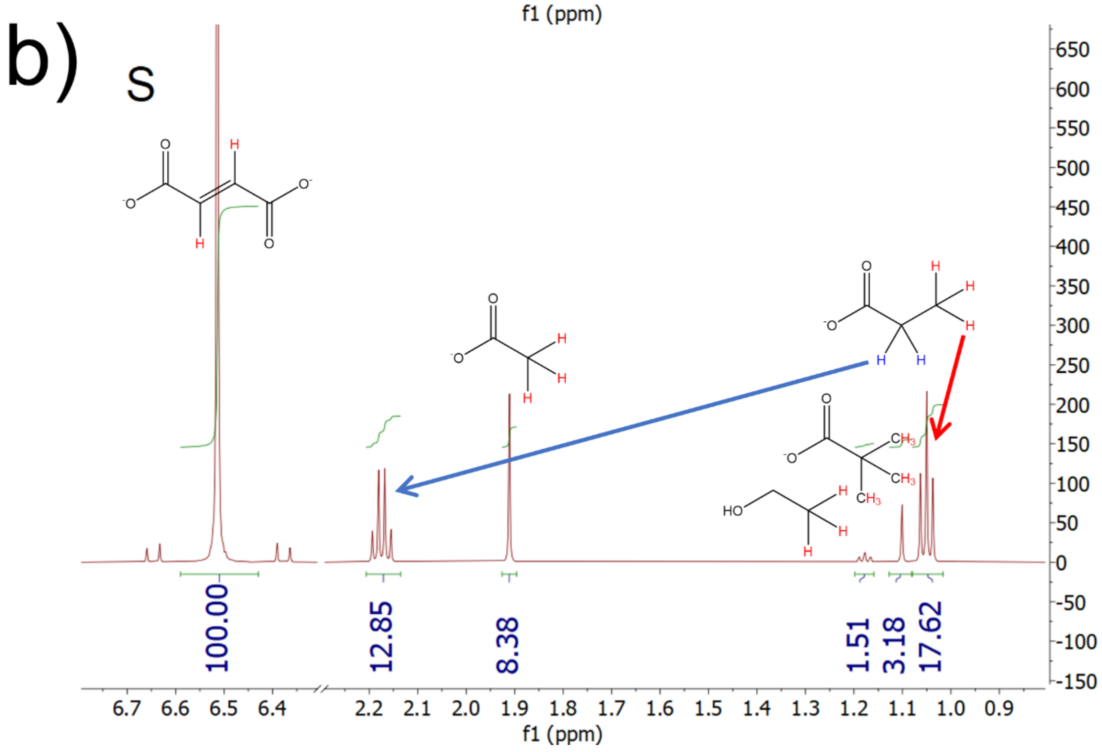
b)
S
100.00
12.85
8.38
1.51
3.18
17.62
f1 (ppm)

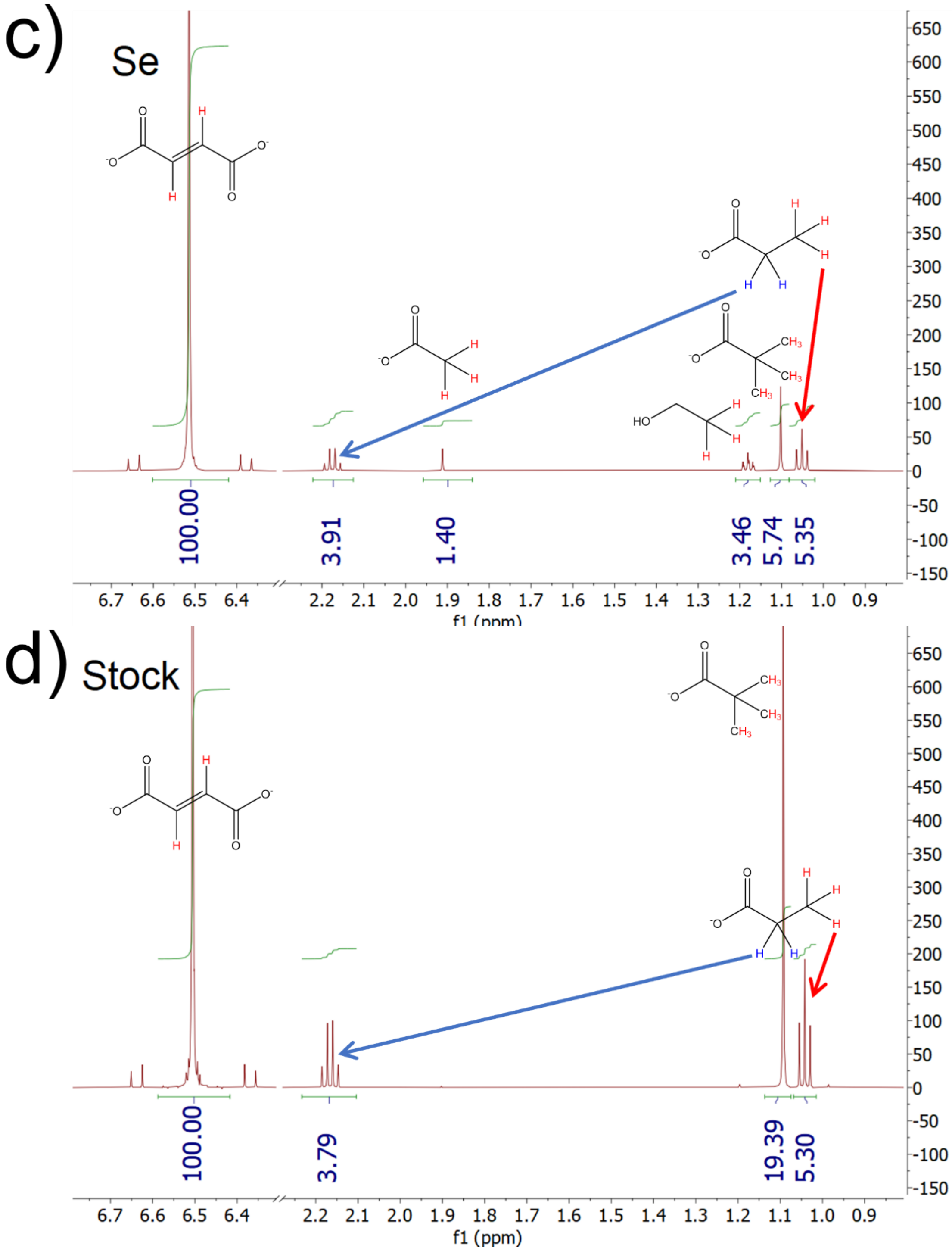

Figure S6: NMR spectra of Keplerates precipitated from a mixture of propionic and pivalic acids and disintegrated by addition of LiOH (a-c) and of the initial mixture of acids (d). Peaks are shown next to their assignment, with assigned hydrogens highlighted with colour. Fumaric acid, whose shift is considerably downfield of the other shifts present, is shown on a separate axis. All integrations are normalised to 100 for the fumaric acid signal occurring at 6.5 ppm. Integrations are calculated excluding ^{13}C satellites.

7. Infrared spectroscopy (FT-IR)

The FT-IR spectra were collected in transmission mode using a Nicolet iS5 FTIR Spectrometer. Compared to the original Keplerate, {$Mo_{132}O_{60}$}, the selenium-based species {$Mo_{132}Se_{60}$} exhibit very similar set of peaks in the IR region located between 750 and 1000 cm^{-1} where Mo^{V}=O, Mo^{VI}=O and Mo^{VI}-O peaks reside. Symmetric and antisymmetric acetate stretches appear significantly stronger for the {$Mo_{132}Se_{60}$} but are shifted by about 20 cm^{-1} in the positive direction for the asymmetric stretch, and 10 cm^{-1} in the negative direction for the symmetric stretch. A pair of peaks at around 1030 cm^{-1} is considerably strengthened and negatively shifted about 30 cm^{-1} (Figures S7 and S8). The purity of the synthesised species has been confirmed further by PXRD and thermogravimetric analyses as shown in Figures S9 and S10.

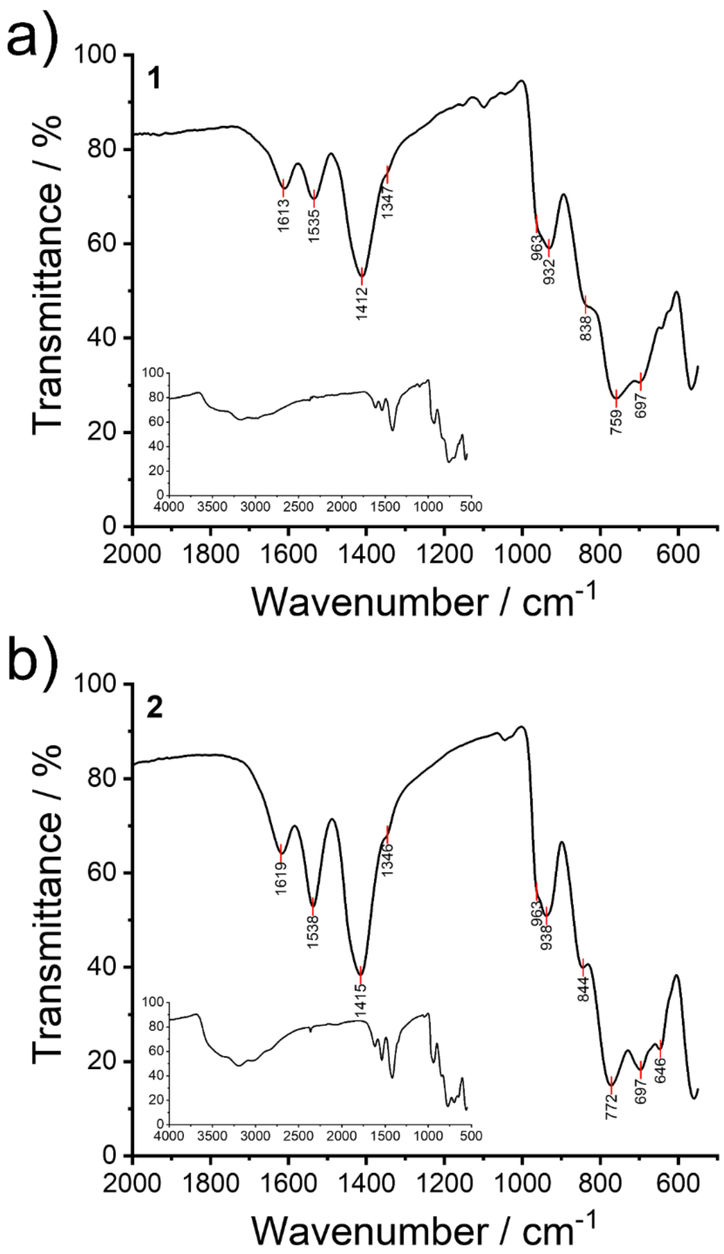

Figure S7: Infra-red spectra of a) **1** and b) **2** between 500 and 2000 cm^{-1} and between 500 and 4000 cm^{-1} (inset). Peaks of **1** located at 1613 cm^{-1} (w), 1535 cm^{-1} (w), 1412 cm^{-1} (s), 1347

cm^{-1} (sh), 963 cm^{-1} (sh), 932 cm^{-1} (s), 838 cm^{-1} (sh), 759 cm^{-1} (m) and 697 cm^{-1} (m). Peaks of **2** located at 1619 cm^{-1} (w), 1538 cm^{-1} (m), 1415 cm^{-1} (s), 1346 cm^{-1} (sh), 963 cm^{-1} (sh), 938 cm^{-1} (s), 844 cm^{-1} (sh), 772 cm^{-1} (s), 697 cm^{-1} (m) and 646 cm^{-1} (w). s = strong, m = medium, w = weak, sh = shoulder. Peaks of **1** (**2**) at 1535 cm^{-1} (1538 cm^{-1}) and 1412 cm^{-1} (1415 cm^{-1}) assigned to acetate C=O stretch (asymmetric and symmetric stretches), peak at 963 and 932 cm^{-1} (963 and 938 cm^{-1}) assigned to Mo=O stretches (Mo^{VI} (W^{VI}) and Mo^{V}).

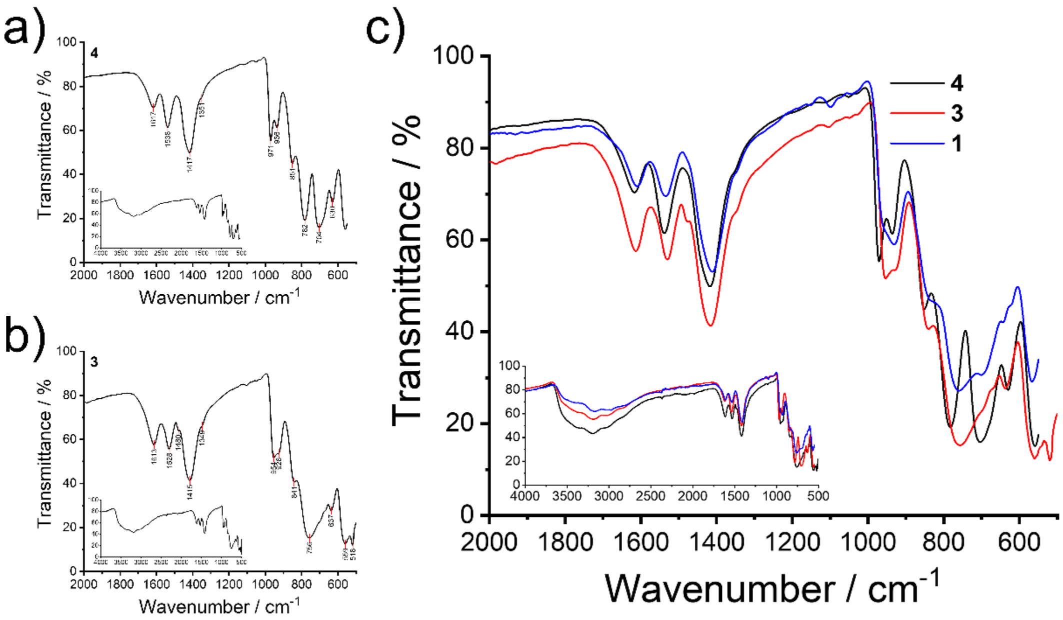

Figure S8: Infra-red spectra of a) **4**, b) **3**, and c) combined spectra of **4**, **3**, and **1** between 500 and 2000 cm^{-1} and between 500 and 4000 cm^{-1} (inset). Peaks of **4** located at 1617 (w), 1538 (m), 1417 (s), 1351 (sh), 971 (s), 936 (s), 851 (m), 782 (s), 704 (s), and 630 cm^{-1} (w), respectively. Peaks of **3** located at 1613 (m), 1528 (m), 1480 (sh), 1415 (s), 1349 (sh), 954 (s), 926 (sh), 841 (m), 756 (s), 637 (w), 559 (w) and 518 cm^{-1} (w), respectively. s = strong, m = medium, w = weak, sh = shoulder. Peaks of **1** as previously described, peaks of **3** and **4** are typical for previously reported $Mo_{132}S_{60}$[4] and $Mo_{132}O_{60}$[5] type Keplerates.

8. Crystal structure data

Suitable single crystals were selected and mounted onto a rubber loop using Fomblin oil. Single-crystal datasets and unit cells were collected at 150(2) K on a Rigaku XtaLAB Synergy R HyPix-Arc diffractometer equipped with a graphite monochromator ($\lambda_{Mo\text{-}K\alpha}$ = 0.71073 Å) on a microfocus X-ray source of rotating anode (50 kV, 24 mA). Data collection and reduction were performed using CrysAlisPro software package. Structure solution and refinement were carried out by using SHELXT-2018/3 and SHELXL-2018/3[6,7] on WinGX platform.[8] Most of the non-hydrogen atoms (including those disordered) were anisotropically refined. Corrections for incident and diffracted beam absorption effects were applied using analytical numeric absorption correction on multifaceted crystal models.[9] CCDC 2226206-2226207 contain the supplementary crystallographic data for compound reported in this paper and can be obtained free of charge via www.ccdc.cam.ac.uk./data_request/cif.

Table S1. Crystal data and structure refinement details for compounds **1** and **2**.

Compound code	**1**	**2**
Empirical formula	$C_{60}H_{802}Mo_{132}N_{42}O_{644}Se_{60}$	$C_{60}H_{802}Mo_{60}N_{42}O_{644}Se_{60}W_{72}$
Formula weight	29823.07	36152.59
Temperature (K)	150(2)	150(2)
Crystal system	Cubic	Cubic
Space group	*Fm*-3*m*	*Fm*-3*m*
a (Å)	46.3086(3)	46.7155(4)
Volume (Å^3)	99308.2(19)	101949(4)
Z	4	4
Density calculated (Mg/m^3)	1.995	2.355
Absorption coefficient (mm^{-1})	3.889	11.020
F(000)	56768	65984
θ range for data collection	2.285 to 24.726°.	2.265 to 24.728°.
Index ranges	-54 <= *h* <= 45,<br>-54 <= *k* <= 52,<br>-51 <= *l* <= 54	-54 <= *h* <= 52,<br>-54 <= *k* <= 54,<br>-23 <= *l* <= 52
Reflections collected	77093	55688
Independent reflections	4106 [*R*(int) = 0.0573]	4207 [*R*(int) = 0.0670]
Completeness (θ = 25.242)	99.6 %	99.8 %
Data/restraints/parameters	4106 / 142 / 255	4207 / 154 / 274
Goodness-of-fit on F^2	1.056	1.025
Final *R* indices [I>2σ(I)]	*R*1 = 0.0775, w*R*2 = 0.2346	*R*1 = 0.0557, w*R*2 = 0.1546
R indices (all data)	*R*1 = 0.1043, w*R*2 = 0.2832	*R*1 = 0.0965, w*R*2 = 0.1956
Max/min Δρ (e Å^{-3})	1.68 and -0.86	1.06 and -0.89

9. PXRD

Powder X-ray diffraction was performed on a Rigaku Miniflex benchtop powder X-ray diffraction instrument in order to verify the purity of the material used in ligand exchange experiments. Samples were purified by stirring in water overnight followed by reprecipitation by addition of excess ammonium chloride before measurement.

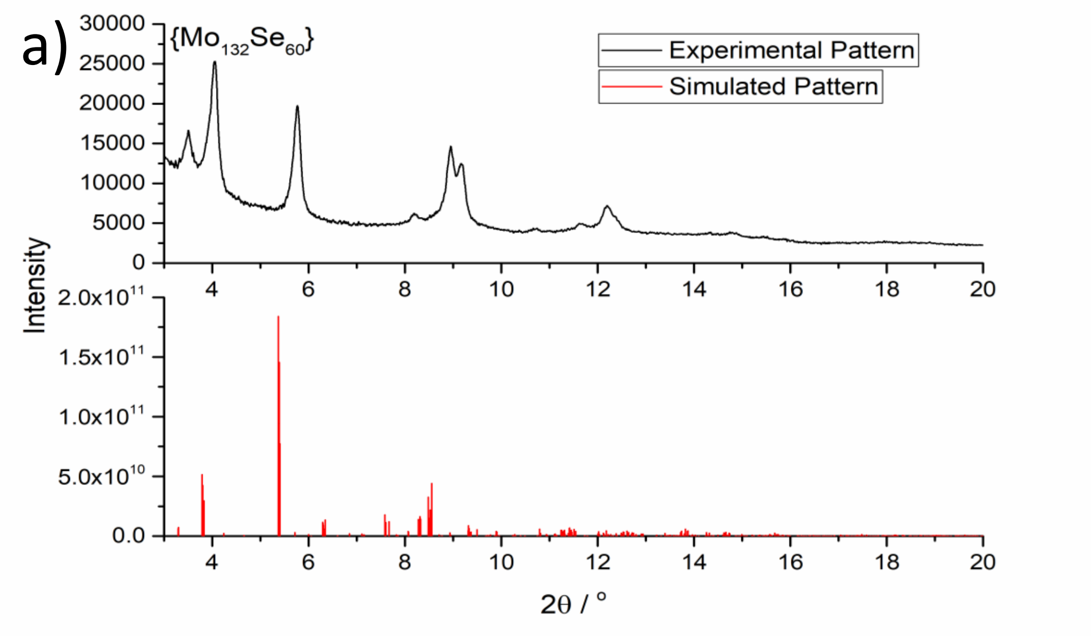

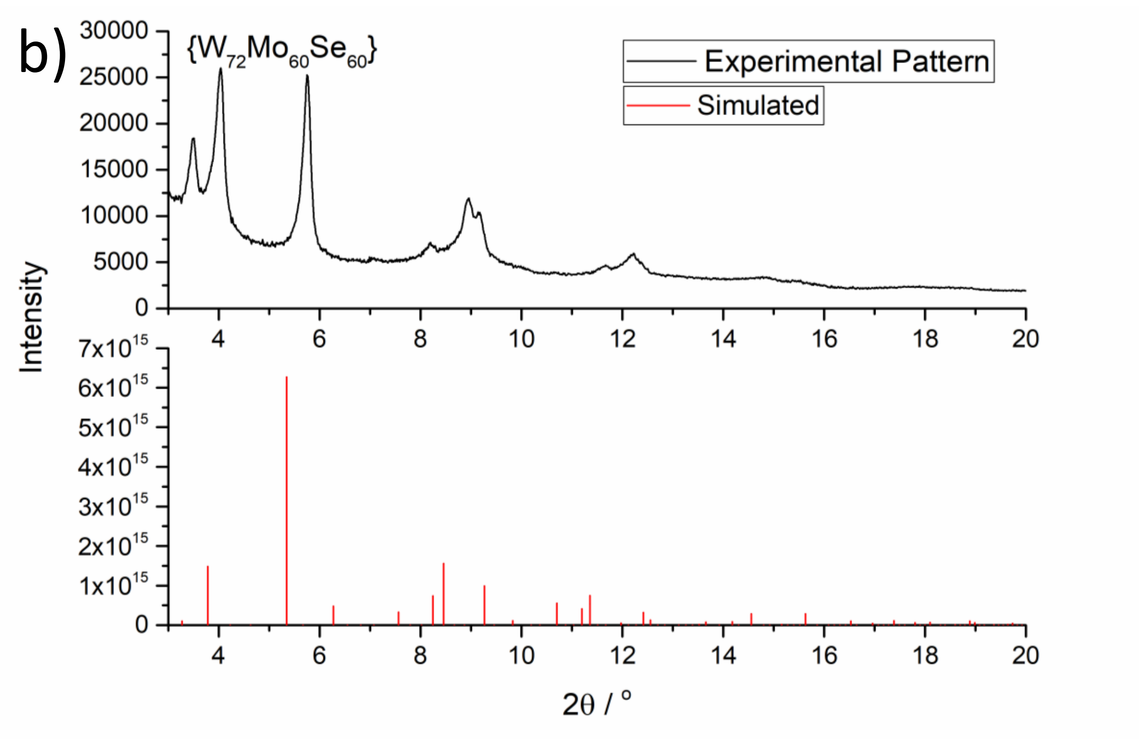

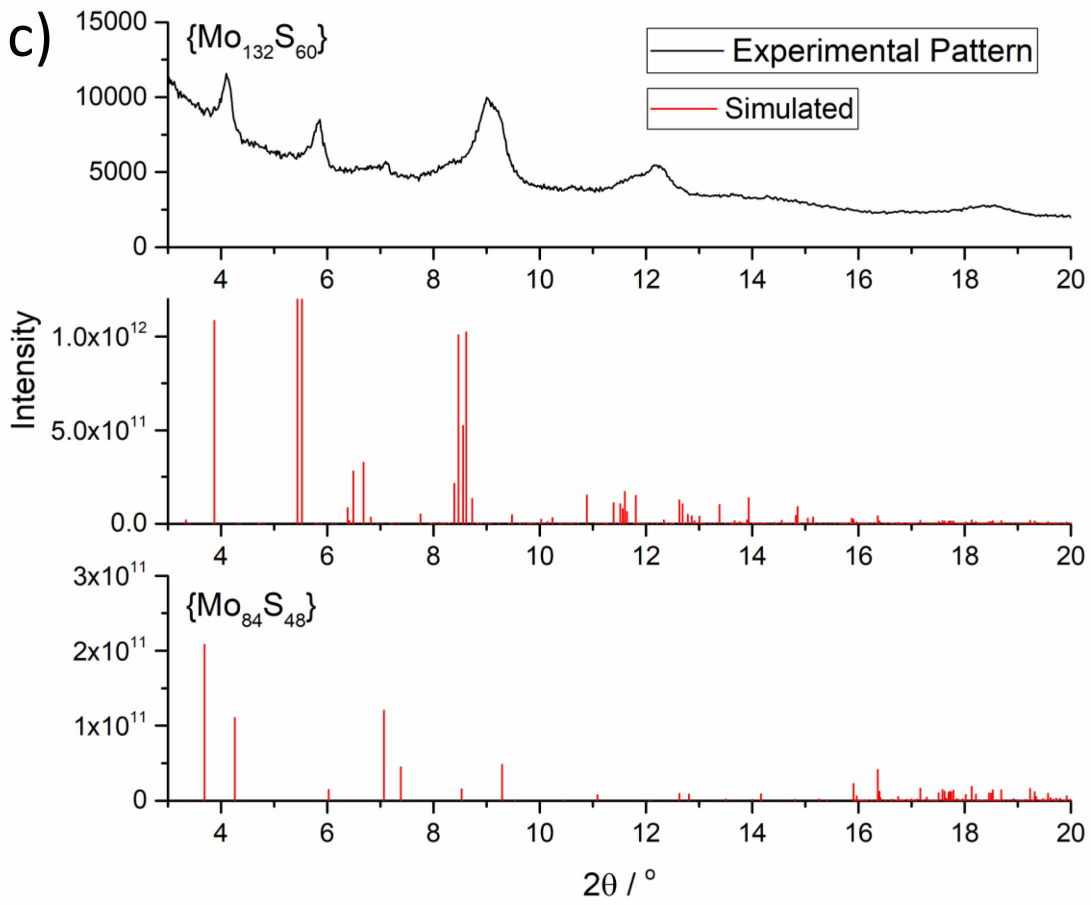

Figure S9: Experimental (black) and predicted (red) PXRD patterns of a) {$Mo_{132}Se_{60}$}, b) {$W_{72}Mo_{60}Se_{60}$} and c) {$Mo_{132}S_{60}$}. Measurements were made up to 40° (2θ) but only showed distinct peaks up to 15°. This is indicative of a lack of small crystalline impurities in high abundance such as sodium or ammonium acetate which may plausibly have crystallised alongside the Keplerate. Similarly, the diffraction peaks match well with those predicted from the single crystal structure indicating that the material is correctly identified by the single crystal structures reported. Previously reported {$Mo_{132}S_{60}$} is formed at similar conditions to an alternative {$Mo_{84}S_{48}$} cube, the predicted pattern of which is included below the predicted pattern of the Keplerate, no evidence of this alternative form is observed in the experimental PXRD.

10. Thermal gravimetric analysis (TGA)

Thermal gravimetric analysis was performed on an SDT Q600 thermal analyser using an alumina pan. Approximately 10 mg of solid material was loaded into the pan which was then heated at a rate of 10°C min^{-1} from room temperature (approximately 20°C) to 800°C under a constant flow of argon gas at 100 mL min^{-1}. The mass loss as a percentage of the original mass was measured.
The size of the weight loss events agrees with the expected chemical formula which is summarised in the table below. The additional 6% of weight which is retained by **2** is additional evidence for the presence of tungsten within the structure. The anticipated chemical formula is matched to the measured weight loss events in the table below.

	Solvent	Organics		Selenium	Remnant	
**1**	Water	Acetate	TMA	Selenium	$MoO_{2.5}$	MoO_3
Number in formula	272	30	14	60	60	72
Predicted percentage	15.8	9.06		15.3	59.8	
Measured percentage	15.0	8.8		15.4	58.1	
	Solvent	Organics		Selenium	Remnant	
**2**	Water	Acetate	TMA	Selenium	$MoO_{2.5}$	WO_3
Number in formula	340	30	26	60	60	72
Predicted percentage	15.5	9.5		11.0	64.2	
Measured percentage	15.3	9.4		12.0	63.1	

Table S2: Summary of the percentage mass of various components of **1** and **2** based on theoretical formula and measured percentage from assignment of the TGA trace. Mass loss events frequently show some overlap such that the predicted percentages may vary from the theoretical by 1-2 percentage points without being in direct conflict. The mass loss events are shown in Figure S9. The remnant mass is the mass remaining at 800°C and is assumed to be made up of molybdenum and tungsten oxides. Additional oxygen atoms may be provided by crystal water. Note that the predicted solvent water and counter cations differ from elemental analysis, given that these represent a small mass fraction minor degrees of overlap of peaks can easily lead to significant errors for these compounds.

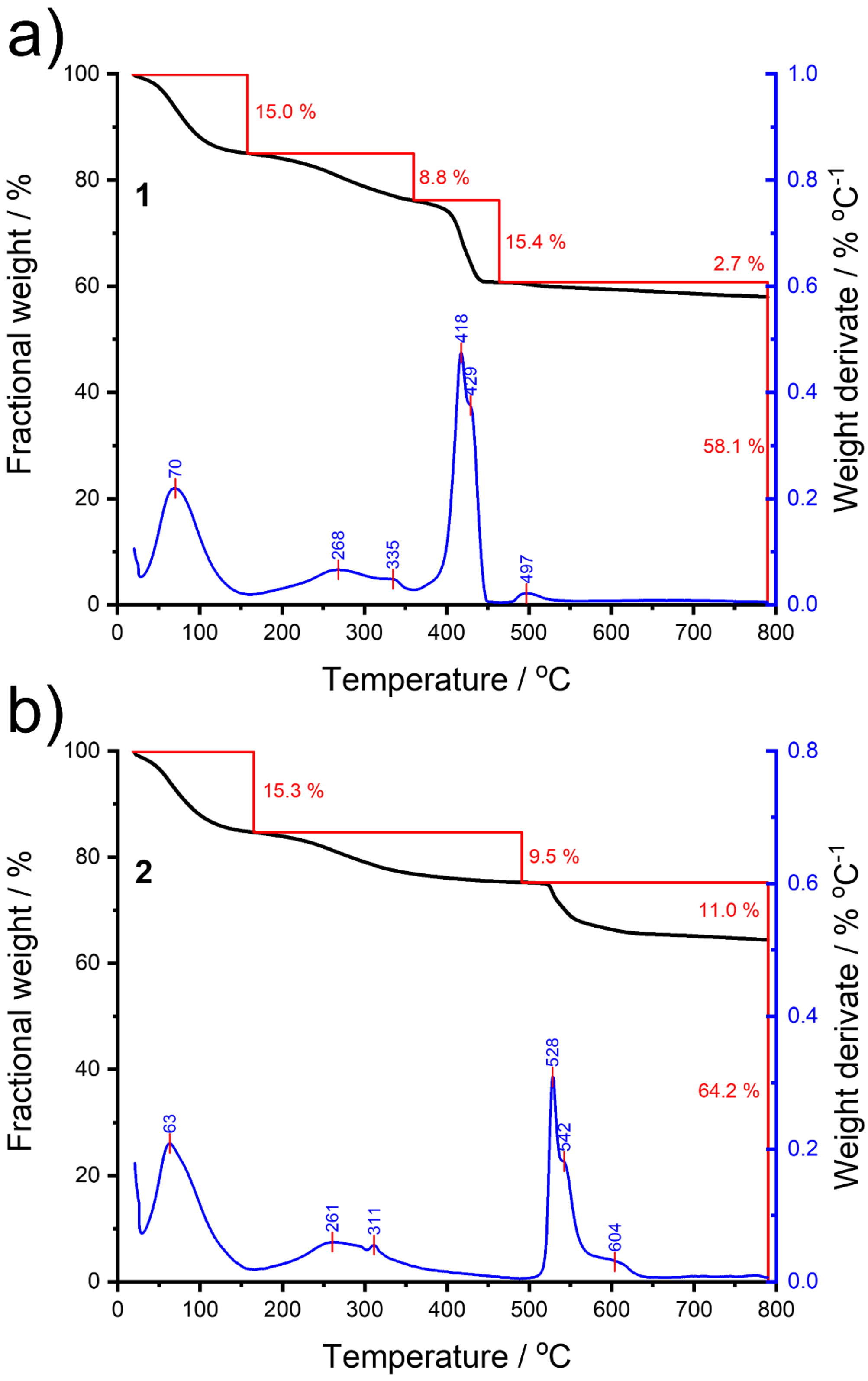

Figure S10: Thermal gravimetric analysis traces of a) **1** and b) **2** heated from 20°C to 800°C. The black line (left axis) shows the fractional change in mass (assumed to be proportional to weight) as a percentage of the initial mass, whilst the blue line indicates the derivative of the mass change with respect to temperature. The red line groups mass loss into distinct events separated by points where the mass loss derivative is essentially zero. The total mass loss of

each event is indicated in red next to the red line, and the final mass as a percentage of the original is also indicated in red. Three main mass loss events occur. The first occurs below 100°C and is assigned to loss of crystal water. The second occurs above 150°C and continues until around 400°C and is attributed to decomposition of organic contents including acetate ligands and tetramethylammonium cations. The final mass loss event occurs at different temperatures for the two traces but features a sharp peak in both cases. This is attributed to loss of selenide. The final mass is considered to consist of a mixture of molybdenum and tungsten oxides.

11. Kinetic model

In order to assess the feasibility of our rationalisation of the kinetic behaviour the competitive exchange with two different ligand types we created a simple model to test if it was possible to recreate the observed behaviour. In particular we wished to show that it was possible for all of the following observations to be simultaneous true when comparing the oxide and sulphide Keplerates: At small mixing times concentrations of pivalic acid were similar or greater in sulphide than oxide, at longer mixing times concentrations of pivalic acid were significantly greater in oxide whilst that rate of change in concentration was greater for sulphide. Simultaneously the relative rates of propanoic acid and pivalic acid exchange should on their own follow the previously observed ordering $Prop_{Oxide}$>$Prop_{Sulphide}$>Piv_{Oxide}>$Piv_{Sulphide}$.

We assume that the exterior concentration is essentially fixed, therefore the rate of change of concentration of ligand inside the capsule can be approximated to follow the form given in equations 1 and 2 for a pair of ligands

$$\frac{d[Ligand_1]}{dt} = a_1\big(1 - b_1([Ligand_1] + [Ligand_2])\big) - (ca_1[Ligand_1]) \quad (1)$$

$$\frac{d[Ligand_2]}{dt} = a_2\big(1 - b_2([Ligand_1] + [Ligand_2])\big) - (ca_2[Ligand_2]) \quad (2)$$

Where a is the rate of exchange through the pore, b is a measure of how strongly the presence of other ligands hinder ligand attachment and is a combination of the degree of steric restriction of the incoming ligand, but also is affected by the total number of sites at equilibrium since the rate of incoming ligand attachment should never become negative (expected to be higher for pivalic acid due to the greater steric bulk, and also observed to be lower for oxide forms leading to more sites being occupied at equilibrium) whilst c measures the rate at which ligands already present detach and return to solution. In the case of a single ligand these equations reduce to an easily solved form which reproduces the exponential behaviour observed in previous experiments.

We simulated 2000-time steps where each step changed the internal concentration of ligand by an amount equal to the rate determined through the previous equations. We were able to recreate this behaviour using the following parameters which obey the constraints described earlier.

	a_{Prop}	a_{Piv}	b_{Prop}	b_{Piv}	c
Oxide	4	0.1	0.04	0.8	0.05
Sulphide	1	0.08	0.06	0.1	0.05

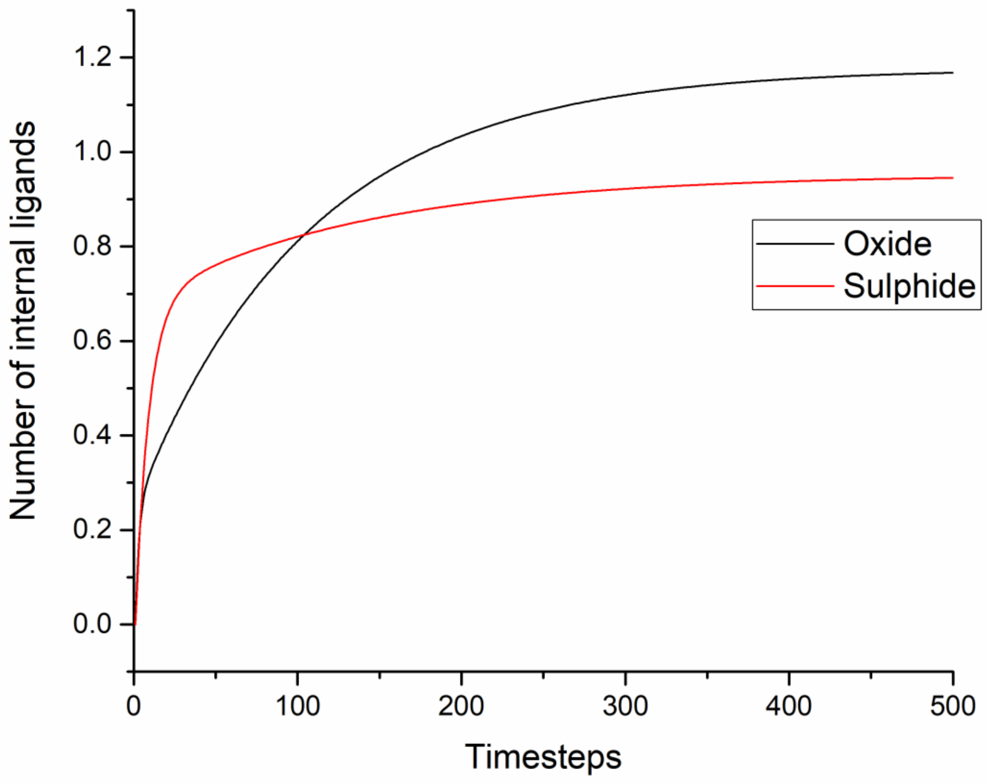

Supplementary Figure 11: Model of behaviour of pivalic acid exchange in oxide and sulphide Keplerates when competing with a more rapidly exchanging propionic acid. The model predicts a more rapid increase in the number of pivalic acid present within the sulphide despite slower transport through pores as a result of the uptake of propionic acid being also slower. Oxide then rapidly overtakes sulphide however, reaching a significantly increased equilibrium position. After 2000 timesteps the net rate of pivalic acid exchange is 8.26×10^{-10} in sulphide, more than ten times the net rate of exchange of 6.05×10^{-11} in oxide.

12. References

[1] F. Bannani, S. Floquet, N. Leclerc-Laronze, M. Haouas, F. Taulelle, J. Marrot, P. Kögerler, E. Cadot, *J. Am. Chem. Soc.* **2012**, *134*, 19342–19345.

[2] A. Müller, E. Krickemeyer, H. Bögge, M. Schmidtmann, F. Peters, *Angew. Chem. Int. Ed.* **1998**, *37*, 3359–3363.

[3] A. Elliott, J. McAllister, L. Masaityte, M. Segado-Centellas, D.-L. Long, A. Y. Ganin, Y.-F. Song, C. Bo, H. N. Miras, *Chem. Commun.* **2022**, *58*, 6906–6909.

[4] F. Bannani, S. Floquet, N. Leclerc-Laronze, M. Haouas, F. Taulelle, J. Marrot, P. Kögerler, E. Cadot, *J. Am. Chem. Soc.* **2012**, *134*, 19342–19345.

[5] A. Müller, E. Krickemeyer, H. Bögge, M. Schmidtmann, F. Peters, *Angew. Chem. Int. Ed.***1998**, *37*, 3359–3363.

[6] G. M. Sheldrick, *Acta Crystallogr. C Struct. Chem.* **2015**, *71*, 3–8.

[7] G. M. Sheldrick, *Acta Crystallogr. A* **2015**, *71*, 3–8.

[8] L. J. Farrugia, *J. Appl. Crystallogr*. **1999**, *32*, 837–838.

[9] R. C. Clark, J. S. Reid, *Acta Crystallogr. A* **1995**, *51*, 887–897.
